# Supplementary material for: Hippocampal place cells encode global location but not connectivity in a complex space
Source: Curr Biol. 2021 Mar 22;31(6):1221–1233.e9. doi: 10.1016/j.cub.2021.01.005 (PMC7988036; doi:10.1016/j.cub.2021.01.005)
Supplement: Document S1. Figures S1–S7 and Tables S1–S4 [file mmc1.pdf]

Current Biology, Volume *31*

Supplemental Information

Hippocampal place cells encode global

location but not connectivity in a complex space

Éléonore Duvelle, Roddy M. Grieves, Anyi Liu, Selim Jedidi-Ayoub, Joanna Holeniewska, Adam Harris, Nils Nyberg, Francesco Donnarumma, Julie M. Lefort, Kate J. Jeffery, Christopher Summerfield, Giovanni Pezzulo, and Hugo J. Spiers

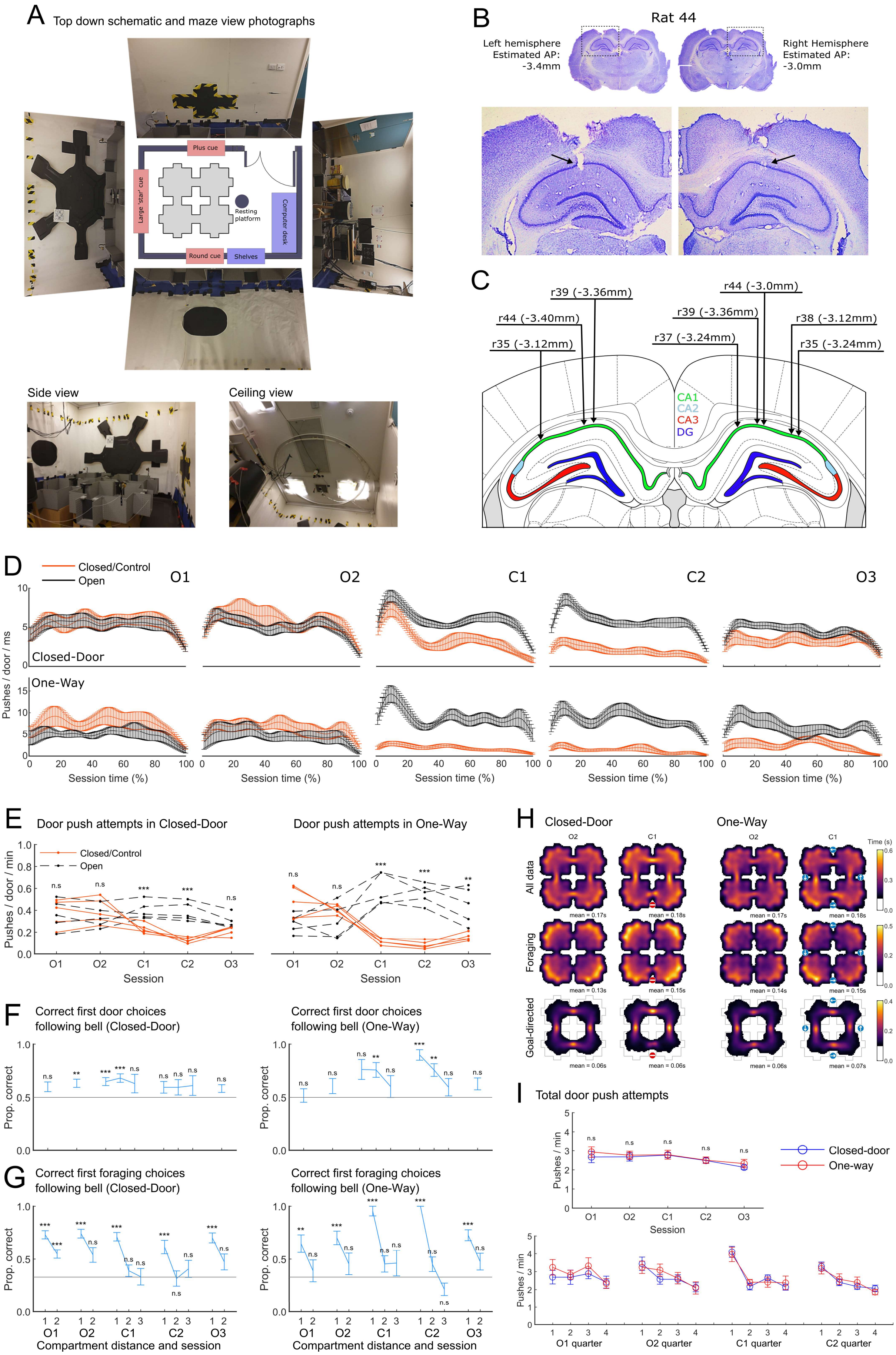

A Top down schematic and maze view photographs
Plus cue
Large 'star' cue
Resting platform
Computer desk
Round cue
Shelves
Side view
Ceiling view
B
Rat 44
Left hemisphere
Estimated AP:
-3.4mm
Right Hemisphere
Estimated AP:
-3.0mm
C
r39 (-3.36mm)
r44 (-3.40mm)
r35 (-3.12mm)
r44 (-3.0mm)
r39 (-3.36mm)
r37 (-3.24mm)
r38 (-3.12mm)
r35 (-3.24mm)
CA1
CA2
CA3
DG
D
Closed/Control
Open
O1
O2
C1
C2
O3
Closed-Door
One-Way
Pushes / door / ms
Session time (%)
E
Door push attempts in Closed-Door
Door push attempts in One-Way
Pushes / door / min
Session
F
Correct first door choices following bell (Closed-Door)
Correct first door choices following bell (One-Way)
Prop. correct
G
Correct first foraging choices following bell (Closed-Door)
Correct first foraging choices following bell (One-Way)
Compartment distance and session
H
Closed-Door
One-Way
All data
Foraging
Goal-directed
Time (s)
I
Total door push attempts
Pushes / min
Closed-door
One-way
O1 quarter
O2 quarter
C1 quarter
C2 quarter

Figure S1: Experimental Room, histology and supplementary behavioural results, related to Figure 1. Here and elsewhere, error bars indicate standard error of the mean and *:$p < .05$, **: $p < .01$, ***: $p < .001$. **(A)** (Top) Top down schematic of the experimental room. The maze is shown in grey. Surrounding this schematic are photos taken from the perspective of the maze along each major axis. (Bottom) Photographs of the maze and ceiling. **(B)** (Top) histological slices for one example rat showing the position of the left and right hemisphere electrodes. Text gives the estimated position of the electrodes relative to Bregma in the anterior-posterior (AP) axis. (Bottom) Close-up view of the dotted region. Arrows denote the position where the electrodes crossed the CA1 cell layer. **(C)** Brain atlas section with superimposed electrode angles and positions estimated for all animals (black arrows). Arrowheads give the estimated position at which the electrodes crossed the CA1 layer. Text gives the rat number and estimated AP position of the electrode tract. Note that the brain atlas section is for an AP of -3.84mm relative to Bregma (the target AP). **(D)** Fine timescale illustration of door-pushing behaviour, showing the kernel-smoothed density estimate of number of door pushes per door side and millisecond for 100 bins per session, averaged over sessions. Profiles appear similar that on larger timescales. **(E)** Door pushes organised as in Figure 1D&E but averaged by rat instead of sessions. Statistics can be seen Table S2. Results are similar to session-averaged plots, indicating reproducibility across rats. **(F, G)** Supplement to figure in Figure 1L&M but separated by session type, averaged across sessions. Statistics can be seen in Table S2. Values are separated by distance between the current box and the goal box (distance of 1 = adjacent box). Distances of 2 are only relevant when a door is locked as both doors are optimal in open sessions and optimal distances of 3 are only available when a door was locked. No noticeable difference can be seen between the two sequence types. **(H)** Occupancy maps, i.e. average time spent in each bin, averaged across sessions for O2 and C1. Before averaging, maps were rotated so that the closed/control door was always found in the south position (in Closed-Door) or reflected around the y-axis so that all doors were locked in a clockwise direction (in One-Way). (Left) Occupancy in the Closed-Door sequence for all data (top), foraging data (middle) and goal-directed data (bottom). (Right) Same for One-Way. **(I)** (Top) Average total door push attempts per session, separated by sequence type. All pushes, whether on open or locked doors, are included. There was an effect of session but not of sequence type. With Closed-Door and One-Way combined, O2 had significantly more pushes than C2 or O3, possibly indicating decreased motivation with time. (Bottom) Same, but separated by session quarters; O3 is not shown. There was no effect of group (Closed-door vs One-Way; Table S3) so all sequence types were combined for further analysis, where a general pattern of decreasing number of door pushes with time for each session was found, except for the first session. This probably indicates a heightened exploration in the first quarter followed by decreased exploration and/or fatigue. See also Supplementary Video 1 videos of door-pushing behaviour and trajectories within the maze.

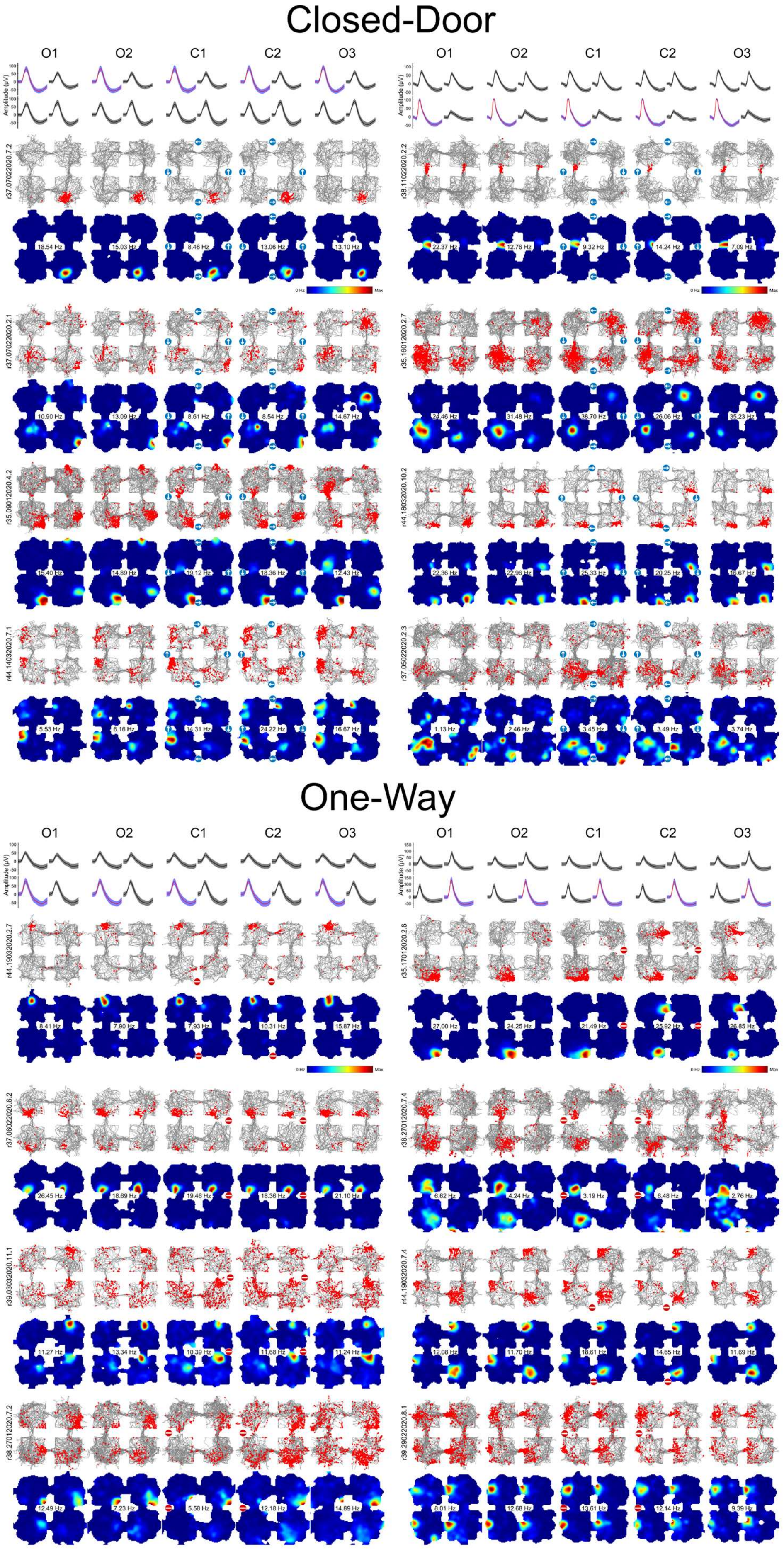
Closed-Door
O1 O2 C1 C2 O3
O1 O2 C1 C2 O3
Amplitude (µV)
r37.07022020.7.2
18.54 Hz 15.03 Hz 8.46 Hz 13.06 Hz 13.10 Hz
r38.11022020.2.2
22.37 Hz 12.76 Hz 9.32 Hz 14.24 Hz 7.09 Hz
0 Hz Max
r37.07022020.2.1
10.90 Hz 13.09 Hz 8.61 Hz 8.54 Hz 14.67 Hz
r35.16012020.2.7
24.46 Hz 31.48 Hz 38.70 Hz 26.06 Hz 35.23 Hz
r35.09012020.4.2
15.40 Hz 14.89 Hz 19.12 Hz 18.36 Hz 12.43 Hz
r44.18032020.10.2
22.36 Hz 22.96 Hz 25.33 Hz 20.25 Hz 16.67 Hz
r44.14032020.7.1
5.53 Hz 6.16 Hz 14.31 Hz 24.22 Hz 16.67 Hz
r37.05022020.2.3
1.13 Hz 2.46 Hz 3.45 Hz 3.49 Hz 3.74 Hz
One-Way
O1 O2 C1 C2 O3
O1 O2 C1 C2 O3
Amplitude (µV)
r44.19032020.2.7
8.41 Hz 7.90 Hz 7.93 Hz 10.31 Hz 15.87 Hz
r35.17012020.2.6
27.00 Hz 24.25 Hz 21.49 Hz 25.92 Hz 26.85 Hz
0 Hz Max
r37.06022020.6.2
26.45 Hz 18.69 Hz 19.46 Hz 18.36 Hz 21.10 Hz
r38.27012020.7.4
6.62 Hz 4.24 Hz 3.19 Hz 6.48 Hz 2.76 Hz
r39.03032020.11.1
11.27 Hz 13.34 Hz 10.39 Hz 11.68 Hz 11.24 Hz
r44.19032020.7.4
12.08 Hz 11.70 Hz 18.61 Hz 14.65 Hz 11.69 Hz
r38.27012020.7.2
12.49 Hz 7.23 Hz 5.58 Hz 12.18 Hz 14.89 Hz
r39.29022020.8.1
8.01 Hz 12.68 Hz 13.61 Hz 12.14 Hz 9.39 Hz

Figure S2: Variety of place cells responses in the Closed-Door sequence and One-Way sequence, related to Figure 2. First row: average ± standard deviation of waveform amplitudes on each tetrode channel for the first two example cells, the largest amplitude is shown in blue. Below, each row pair shows the spike plots (top: trajectory in grey, spikes in red) or speed-filtered rate maps (bottom: number indicates maximum firing rate; see *Methods - Firing rate maps and spatial information*) for a given place cell recorded in the five sessions of a sequence. Cells were selected to show a range of place field numbers (increasing from 1 at the top to 4 at the bottom) and response types across different rats. Foraging as well as goal-directed data are included. Locked doors or locked door sides are indicated by signs as in Figure 1. Cells had a variety of field numbers, repeating or not. While the spatial activity of place cells remained generally stable across consecutive sessions, place fields sometimes appeared and disappeared in a way not specifically linked to the changes in connectivity. See also Supplementary Video 2 for spike plots and rate maps of all used place cells (foraging + goal-directed).

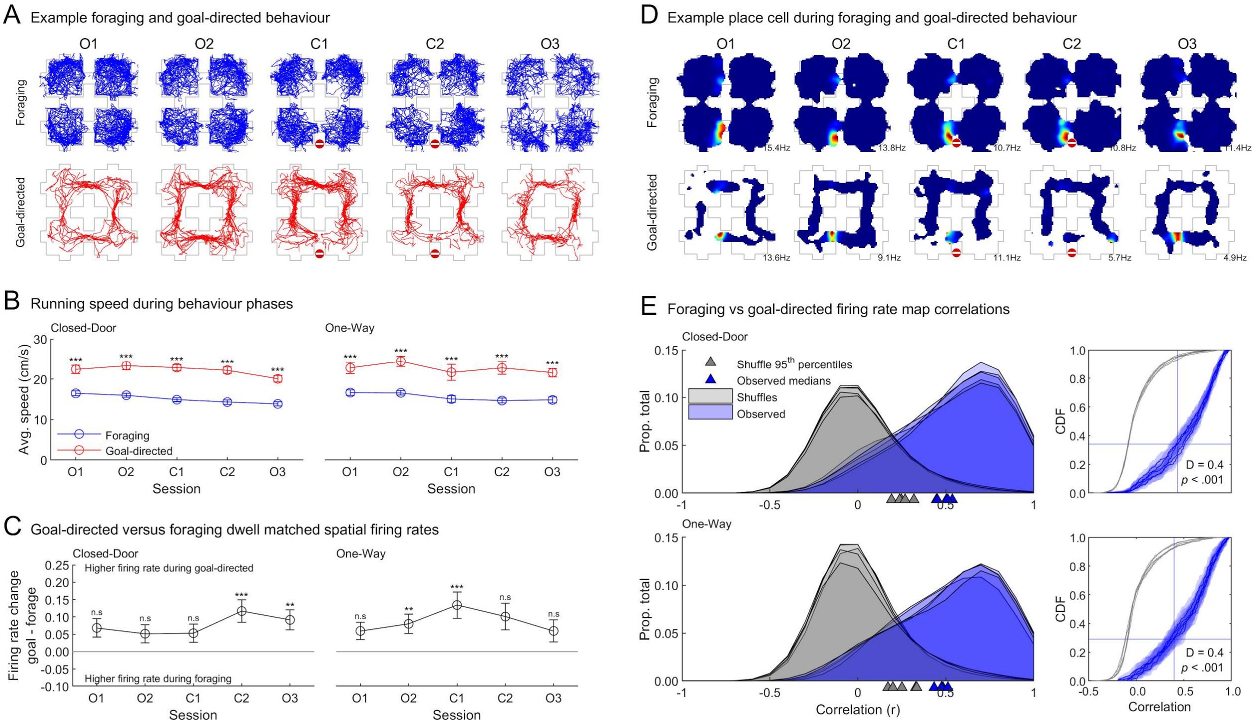

Figure S3: No spatial remapping between foraging and goal-directed epochs, related to Figure 2. See Table S4 for all statistical results. Task remapping has been shown under some conditions, usually if tasks are performed in separate blocks [S1-3], but not when tasks are intermingled within the same experimental session [S4-5]. We compared foraging to goal-directed activity for each session type, see *Methods - Behaviour discrimination*. **(A)** Example of trajectory discrimination between foraging (Top) and goal-directed (Bottom) for one Closed-Door sequence. Note that foraging trajectories are comparable between sessions while goal-directed ones are strongly affected by the door closure; see also Figure S1H for dwell maps averaged over all sessions. **(B)** Average running speed over sessions for the different task phases. Speed during goal-directed movement was always higher. Speed was slower in later sessions, probably indicative of decreasing motivation. **(C)** Comparison of average firing rate between task epochs, computed over common spatial bins as the difference of goal-directed and foraging average firing rates, averaged over place cells. Positive numbers indicate increased firing rate during goal-directed epochs, significantly so for a subset of the sessions (two-sided signed rank tests with Holm-Bonferroni correction). **(D)** Example rate maps of a cell recorded in the same session as A, divided between foraging (Top) and goal-directed (Bottom). Note that the spatial activity of this cell remains stable between the two types of behaviours. Colour scale is the same as previous rate maps, number indicates peak firing rate. See *Methods - Foraging vs goal-directed remapping.* **(E)** (Left) Distributions of the correlations between goal-directed and foraging rate maps for all sessions (O1, O2, C1, C2, O3) in blue and shuffled data in grey. In all sessions, the median of the observed correlations exceeded the 95th percentile of a corresponding chance distribution, demonstrating that the data from both task phases were more similar than chance, indicating no global remapping between the two task phases. (Right) Cumulative distribution function of each group. The 95th percentile of the shuffle distribution (O2) is indicated by a vertical blue line and the intersection of this with the O2 data distribution is indicated by a horizontal line, showing that more than 60% of the cells are more stable than chance; thus, only a minority of cells changed their firing location between foraging and goal-directed phases. (<37% in Closed-Door, <30% in One-Way). Text gives the result of a two-sample Kolmogorov-Smirnov test comparing the two distributions in session O2. There was no effect of connectivity on this (no significant difference between data distributions, all p>.05, Kolmogorov-Smirnov tests, Holm-Bonferroni corrected).

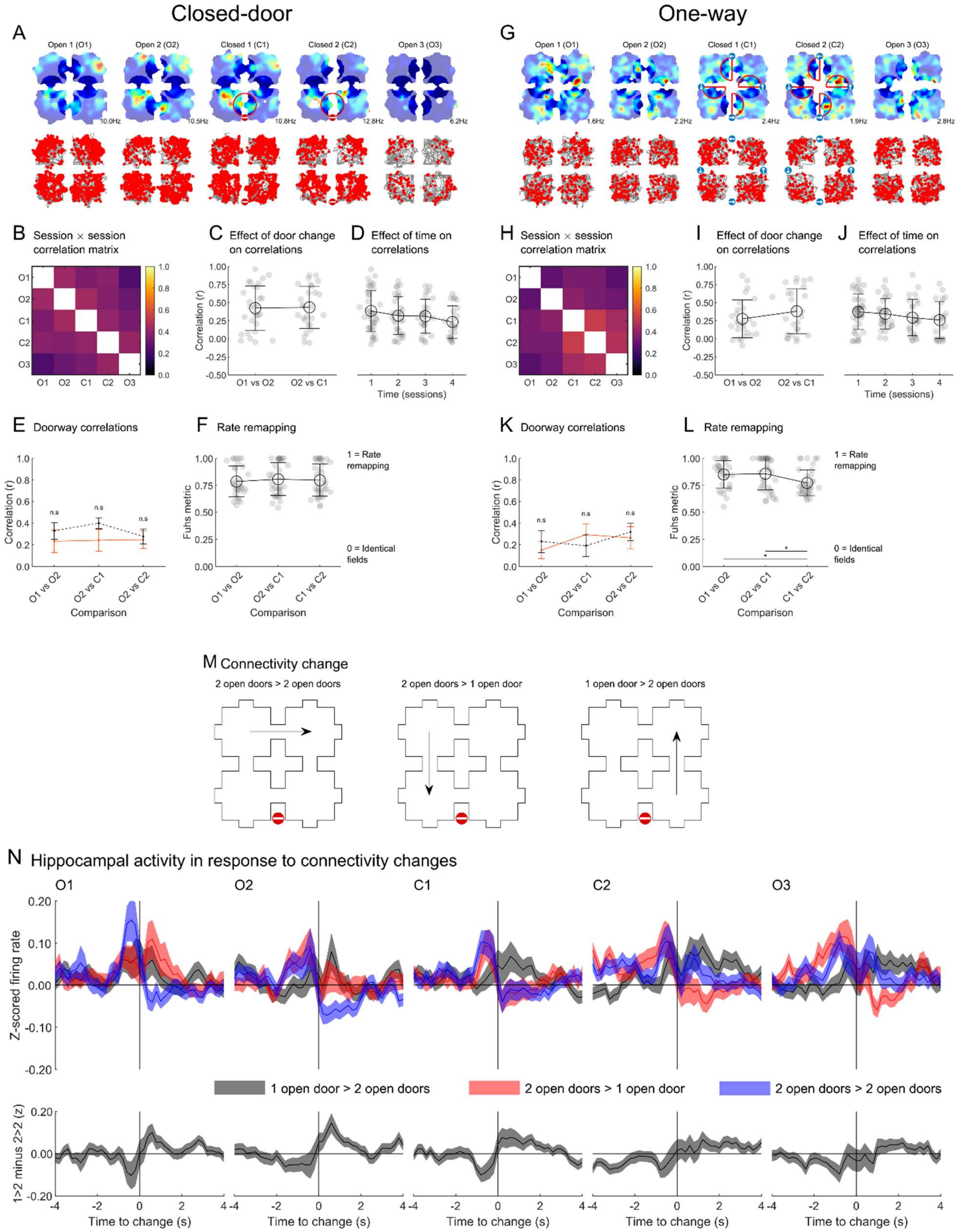

Figure S4: No effect of connectivity changes on pyramidal non-place cells or on average place cell firing rate when crossing doors, related to Figure 2. (**A-L**) Pyramidal non-place cells were analysed in the same was as place cells in Figure 2, with A-F related to the Closed-Door sequence and G-L related to One-Way, legend is the same as Figure 2. (**C**) No effect of session was found, similarly to place cells: F(1,54) = 0.0, *p* = .89. (**D**) No effect of time was found, unlike for place cells F(3,115) = 1.7, *p* = .16. (**F**) There was no effect of connectivity on rate remapping: F(2,98) = 0.2,

p = .85. (**I**) There was no effect of session on cross-session correlations: F(1,51) = 1.8, p = .19. (**J**) Again, unlike for place cells, no effect of time on correlations was found: F(3,118) = 1.4, *p* = .26; (**L**) An effect of session was found for rate remapping: F(2,102) = 4.5*, p* = .014; post-hoc tests are as follows: O1O2 vs O2C1: *p* = .97; O1O2 vs C1C2: *p* = .047; O2C1 vs C1C2: *p* = .019; however, these effects are not specific to the connectivity change. In summary, even in this population of non-place cells, no effect specific to changing the connectivity (i.e. when comparing O2 to C1) was found. Interestingly, the effect of time observed in place cells was not significant in this population of non-place cells. In addition, average cross-session correlation values were generally lower than for place cells (Figure 2), indicating less spatially-stable activity in this population. (**M, N**) Human participants entering a street that had greater connectivity than the preceding street had increased activity in their posterior right hippocampus, which decreased if the street had less connectivity [S6]. We tested this here by comparing activity when entering a box depending on the number of transitions available from that box. Z-scores of firing rates were computed for each place cell, binned in 0.2s bins in a time window ranging from -4s to +4s around entry into a box, and averaged over all place cells. No effect was found. (**M**) Schematic showing the possible connectivity transitions available to the rats when one door is closed. Rats can move to a box with equivalent connectedness (Left), diminished connectedness (Middle) or increased connectedness (Right). (**N**) (Top) Mean and SEM firing rates for all place cells around these connectivity transitions, using all data (foraging + goal-directed). (Bottom) Difference between the firing rate profile for increased connectedness and equivalent connectedness. Values higher than 1.96 or lower than -1.96 would represent a significant change in activity. Note that in the open sessions (O1, O2 & O3) all compartments are equally connected so the groups are arbitrary.

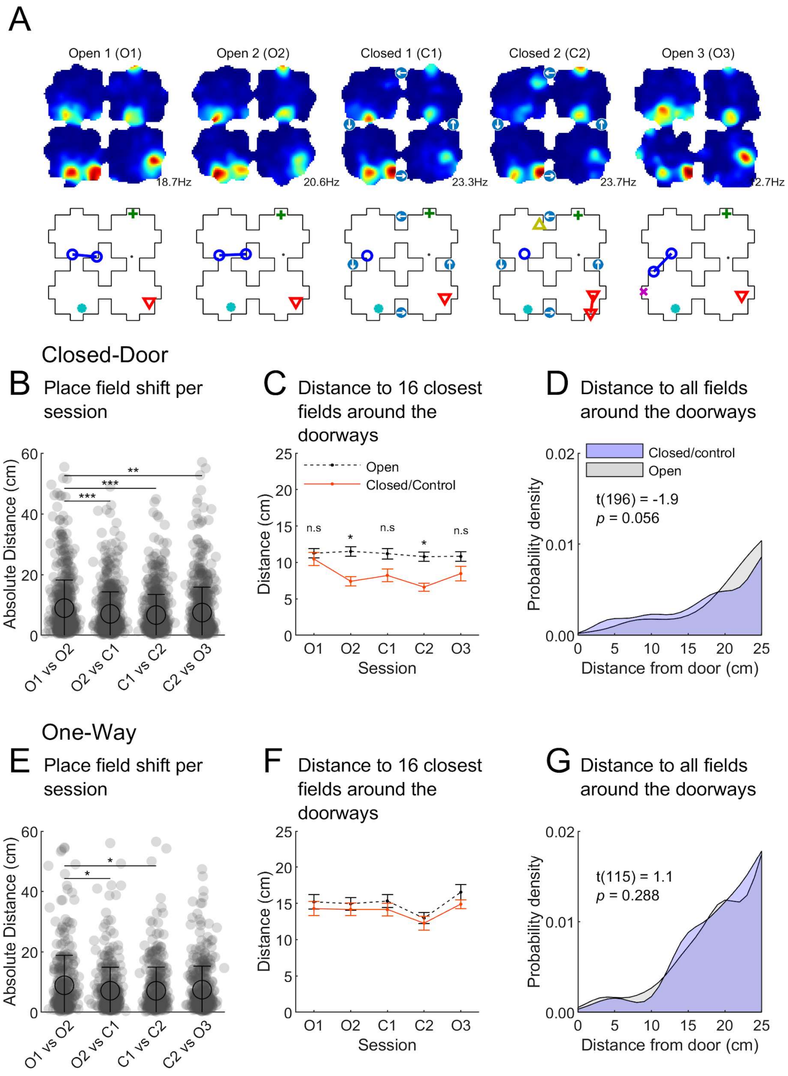

Figure S5: No change in distance of fields to doors with connectivity, related to Figure 3. See Table S4 for statistics and *Methods – Fields distance to doorways*. (**A**) An example place cell recorded across sessions. (Top) Firing rate maps, blue denotes low or no firing, red denotes high firing. Maximum firing rates are given as text. (Bottom) Centroids of place fields detected in each map. Each field is tracked across sessions and represented by the same coloured/shaped marker in each. Where a field was detected to split in one or more sessions, the same marker is joined by a line of the same colour. (**B**) Absolute distance that place field centroids shifted of between consecutive sessions. Place fields moved the most between sessions O1 and O2 indicating no specific field movement due to connectivity change. (**C**) Distance to the closest 16 fields from the doors in each closed-door session. In O2 and C2, fields are significantly closer to the locked doors, although this is a general trend present in almost all sessions, including O2 and O3 where there was no connectivity change. Overall, although there were some differences between the control/locked door and the open doors, these effects were not specific to the closed-door sessions. (**D**) Distance to all fields from the doors in closed-door session C1 only. Text gives the result of a two-sample t-test comparing the distributions. These were not significantly different. (**E-G**) Same as B-D but for the One-Way condition with similar results.

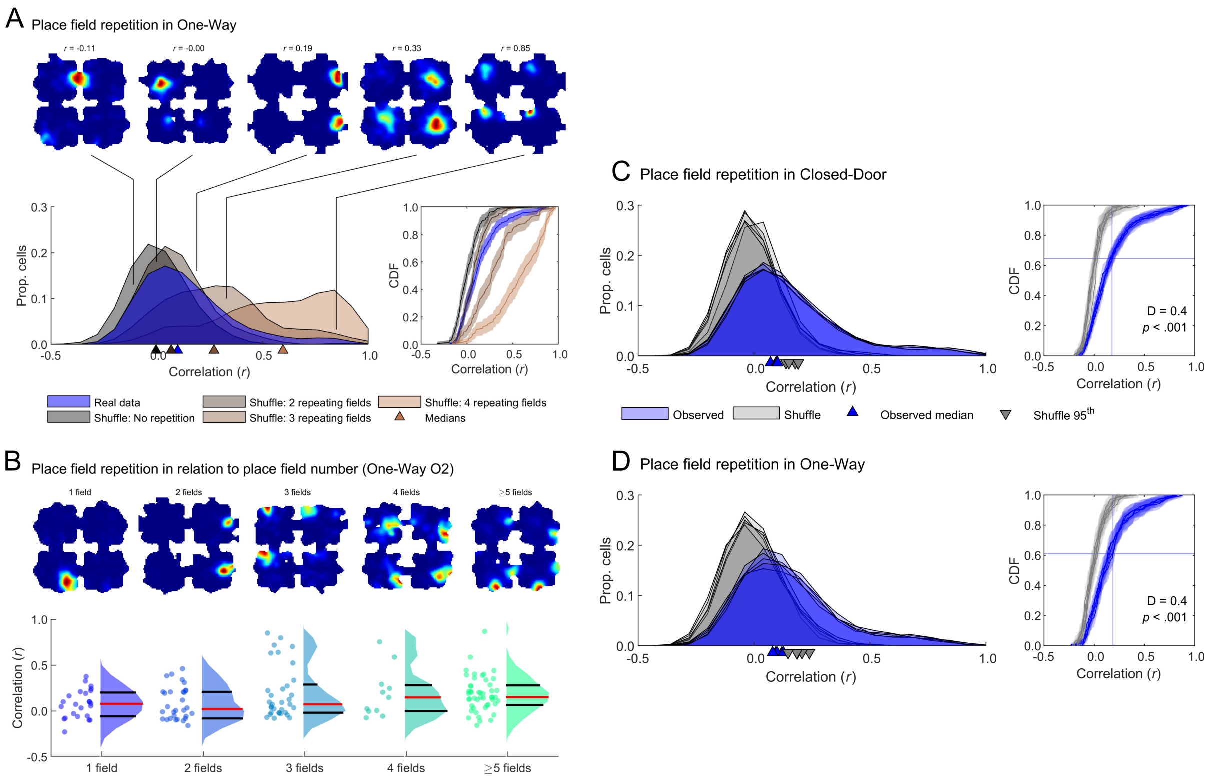

Figure S6: Most place cells encode space globally in One-Way too, independently of connectivity. Related to Figure 4. (**A&B**) Supplement for Figure 4D, E showing data for the One-Way condition; see *Methods - Place field repetition*. (**A**) (Top) Example rate maps (all data, speed-filtered, red indicates high firing rate, dark blue indicates low firing rate) with indicated correlation values on the O2 data distribution (blue). (Left) Blue distribution represents real data cross-box correlation values for session O2. All other distributions represent different shuffles designed to simulate the correlations expected if cells did not have any repeating fields or repeated fields in 2, 3 or 4 compartments. Medians for all distributions are indicated by corresponding markers along the x-axis. (Right) Same data as left but showing the empirical cumulative distribution functions, shaded areas represent lower and upper confidence intervals. Results are similar to Closed-Door with average cross-box correlation values most similar to a situation with 2 repeating fields. (**B**) Same as Figure 4E for One-Way (O2 only). (Top) Example rate maps sorted by the number of detected place fields. Bottom: box correlations scores also separated by number of detected fields; dots show individual place cell data, distribution is shown as violin plot, with median in red and quartiles in black. The same bimodality as for Closed-Door emerges for 3 or more fields, showing a small subset of cells with fully-repeating place fields (correlations>0.5). (**C**) (Left) In blue, distributions of cross-box correlation values for all sessions (O1, O2, C1, C2, O3); in grey, distributions of shuffled correlation values for each session. Medians for all distributions are indicated by corresponding markers along the x-axis. Data medians were always below the 95th percentile of the corresponding shuffle, indicating similarity to the shuffle, i.e. low field repetition overall. (Right) Same data but showing the empirical cumulative distribution functions, shaded areas represent lower and upper confidence intervals. Vertical blue line indicates the 95th percentile of the shuffle distribution (for O2) and its intersection with the data distribution is indicated by a horizontal line, showing that the majority of cells do not repeat more than chance. Text gives the result of a two-sample Kolmogorov-Smirnov test comparing the two distributions for O2. Data distributions from different session types did not differ from each other (p>.05 in all cases, two-sample Kolmogorov-Smirnov tests), in line with our other results finding no effect of connectivity changes. (**D**) Same as C but for One-Way.

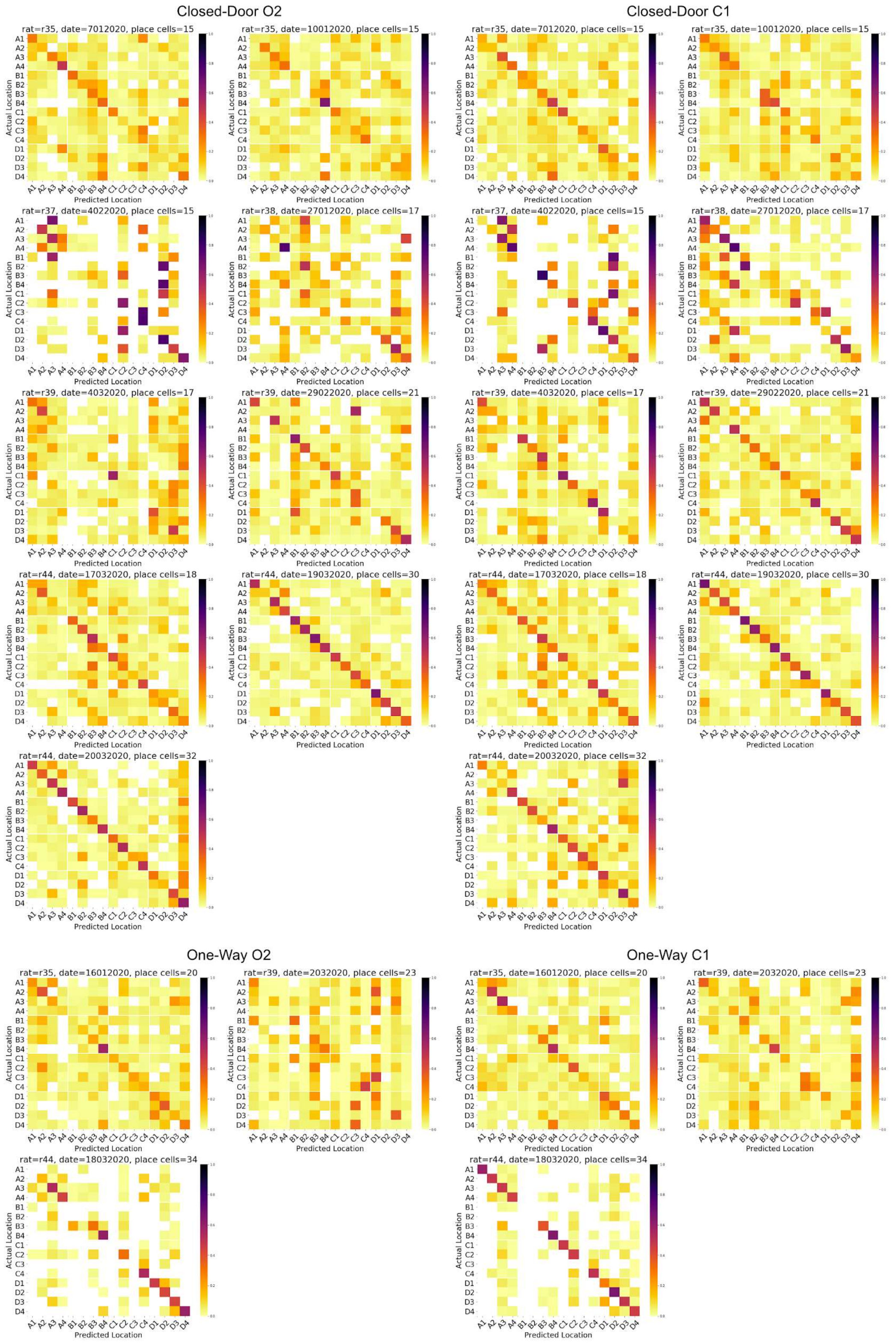

Figure S7: Individual confusion matrices for all sessions used in the quadrant Bayesian decoding analysis, related to Figure 4F-H. See *Methods - Bayesian decoding of box quadrants.* Only sessions with at least 15 simultaneously recorded place cells were used. The diagonal pattern can be observed for most sessions, with some exceptions for those with lower numbers of cells (indicated in the title of each plot).

	Number of full sequences		Place cells (% of all neurons)		Place fields	
Rat	Closed-Door	One-Way	Closed-Door	One-Way	Closed-Door	One-Way
r35	6	3	73 (74.5)	43 (75.4)	525	267
r37	3	2	29 (80.6)	26 (83.9)	91	95
r38	4	2	32 (94.1)	12 (70.6)	93	53
r39	3	2	47 (79.7)	35 (58.3)	254	358
r44	3	2	80 (81.6)	45 (67.2)	412	236
Total	19	11	261 (80.3)	161 (69.4)	1375	1009

Table S1: Numbers of sequences, place cell and place field numbers per rat and condition, related to Figure 1 and 2. Note that place field numbers used the session with the highest mean firing rate (used for place cell categorisation), thus, they represent an upper bound and are not representative of average sessions.

Condition	Isolation distance	L ratio	Waveform height (μV)	Waveform width (μs)	Mean firing rate (Hz)
Closed-Door	23.6 ± 24.2	0.03 ± 0.06	48.4 ± 39.5	485.1 ± 65	0.73 ± 0.76
One-way	20.2 ± 13.4	0.04 ± 0.1	45.5 ± 32.4	481.9 ± 73	0.65 ± 0.63

Table S2: Cluster quality measures for putative place cells, related to Figure 2. See *Methods - Cluster quality measures* for detailed methods regarding these metrics. Values are mean ± standard deviation

Panel and dependent variable	Omnibus test	Effect of group (E-G: closed vs open; I: Closed-Door or One-Way)	Effect of session (O1, O2, C1 …) or quarters (I with sequences combined)	Interaction groupXsession or group X quarters (I bottom)	Post-hoc tests across groups (corrected)
E<br>Closed-Door, pushes/door/min	Two-way repeated measures ANOVA	F(1,16) = 13.5, *p* = .021	F(4,16) = 5.3, *p* = .006	F(4,16) = 27.1, *p* < .001	C1: *p* < .001<br>C2: *p* < .001<br>All other *p* > .90
E<br>One-Way, pushes/door/min		F(1,16) = 20.2, *p* = .011	F(4,16) = 3.2, *p* = .041	F(4,16) = 26.4, *p* < .001	C1: *p* < .001<br>C2: *p* < .001<br>O3: *p* = .006<br>All other *p* > .90
F<br>Correct first door choices post-bell, Closed-Door	Independent one-sample t-tests with correction	Compared to chance (1/2):<br>From left to right: *p* > .99, *p* = .003, p < .001, p < .001, *p* > .99, *p* > .99, *p* > .99, *p* > .99, *p* > .99			
F<br>Correct first door choices post-bell, One-Way		Compared to chance (1/2):<br>From left to right: *p* > .99, *p* > .99, *p* > .99, *p* = .004, *p* > .99, *p* < .001, *p* = .001, *p* > .99, *p* > .99			
G<br>Correct first foraging post-bell, Closed-door		Compared to chance (1/3):<br>From left to right: *p* < .001, *p* < .001, *p* < .001, *p* > .99, *p* < .001, *p* > .99, *p* > .99, *p* < .001, *p* > .99, *p* > .99, *p* < .001, *p* > .99			
G<br>Correct first foraging post-bell, One-Way		Compared to chance (1/3):<br>From left to right: p = .003, p > .99, p < .001, p > .99, p < .001, p > .99, p > .99, p < .001, p > .99, p > .99, p < .001, p > .99			
I (Top)<br>Total number of pushes	Two-way repeated measures ANOVA	F(1,140) = 0.7, *p*=.398	F(4,140) = 2.5, *p*=.043	F(4,140) = 0.1, *p*=.98	All *p* > .90
I (Top)<br>Total number of pushes, Closed-Door and One-Way combined	One-way repeated measures ANOVA	N.A.	F(4,145) = 2.8, *p*=.026	N.A.	O1 vs O3: *p*=.048<br>C1 vs O3: *p*=.041<br>All other p>.05
I (Bottom)<br>Total number of pushes, O1	Two-way repeated measures ANOVA	F(1,112) = 0.9, *p* = .34	F(3,112) = 1.4, p = 0.25	F(3,112) = 0.3, *p* = .84	Effect of group in each quarter:<br>All *p* > .90
I (Bottom)<br>Total number of pushes, O2		F(1,112) = 0.1, *p* = .71	F(3,112) = 4.9, *p* = .0031	F(3,112) = 0.4, *p* = .73	Effect of group in each quarter:<br>All *p* > .90
I (Bottom)<br>Total number of pushes, C1		F(1,112) = 0.0, *p* = .91	F(3,112) = 20.4, *p* < .001	F(3,112) = 0.4, *p* = .72	Effect of group in each quarter:<br>All *p* > .90
I (Bottom)<br>Total number of pushes, C2		F(1,112) = 0.0, *p* = .90	F(3,112) = 10.8, *p* < .001	F(3,112) = 0.4, *p* = .74	Effect of group in each quarter:<br>All *p* > .90
I (Bottom)<br>Total number of pushes, Closed-Door and One-Way combined, O1	One-way repeated measures ANOVA	N.A.	F(3,116) = 1.2, p = 0.31	N.A.	N.A.
I (Bottom)<br>Total number of pushes, Closed-Door and One-Way combined, O2		N.A.	F(3,116) = 5.5, *p* < .001	N.A.	Q1 vs Q4:*p*<.001<br>all other p > .05
I (Bottom)<br>Total number of pushes, Closed-Door and One-Way combined, C1		N.A.	F(3,116) = 3.5, *p* < .001	N.A.	Q1 vs Q2,<br>Q1 vs Q3,<br>Q1 vs Q4:<br>*p* < .001<br>all other p > .50
I (Bottom)<br>Total number of pushes, sequences combined, C2		N.A.	F(3,116) = 12.1, *p* < .001	N.A.	Q1 vs Q2:*p*=.0033<br>Q1 vs Q3:*p*<.001<br>Q1 vs Q4:*p*<.001<br>all other p>.10

Table S3: Statistics corresponding to Figure S1. All post-hoc tests were based on estimated marginal means and corrected using the Tukey-Kramer method.

Panel and dependent variable	Omnibus test	Effect of group (closed/control or open)	Effect of session	Interaction group X session	Post-hoc tests across groups (corrected)
B left<br>Foraging vs goal-directed running speed Closed-Door	two-way repeated measures ANOVA	F(1,72) = 156.2, $p < .001$	F(4,72) = 6.8, $p < .001$	F(4,72) = 4.2, $p = 0.042$	All p < .001
B right<br>Foraging vs goal-directed running speed One-Way	two-way repeated measures ANOVA	F(1,40) = 47.5, $p < .001$	F(4,40) = 3.9, $p = 0.0089$	F(4,40) = 1.2, $p = 0.32$	All p < .001
C left<br>Foraging vs goal-directed firing rates One-Way	Independent one-sample t-tests with Holm-Bonferroni correction	Compared to chance (0):<br>From left to right: $p > .99$, $p > .99$, $p > .99$, $p < .001$, $p = .002$			
C right<br>Foraging vs goal-directed firing rates One-Way		Compared to chance (0):<br>From left to right: $p > .99$, $p = .004$, $p < .001$, $p > .99$, $p > .99$			

Table S4: Statistical test results corresponding to Figure S3. Note: all post-hoc tests were based on estimated marginal means and corrected using the Tukey-Kramer method.

Supplemental References

S1. Markus, E.J., Qin, Y.L., Leonard, B., Skaggs, W.E., McNaughton, B.L., and Barnes, C.A. (1995). Interactions between location and task affect the spatial and directional firing of hippocampal neurons. J. Neurosci. *15*, 7079–7094.

S2. Wiener, S.I., Paul, C.A., and Eichenbaum, H. (1989). Spatial and behavioral correlates of hippocampal neuronal activity. J. Neurosci. *9*, 2737–2763.

S3. Spiers, H.J., Olafsdottir, H.F., and Lever, C. (2018). Hippocampal CA1 activity correlated with the distance to the goal and navigation performance. Hippocampus *28*, 644–658.

S4. Trullier, O., Shibata, R., Mulder, A.B., and Wiener, S.I. (1999). Hippocampal neuronal position selectivity remains fixed to room cues only in rats alternating between place navigation and beacon approach tasks. Eur. J. Neurosci. *11*, 4381–4388.

S5. Duvelle, É., Grieves, R.M., Hok, V., Poucet, B., Arleo, A., Jeffery, K.J., and Save, E. (2019). Insensitivity of Place Cells to the Value of Spatial Goals in a Two-Choice Flexible Navigation Task. J. Neurosci. *39*, 2522–2541.

S6. Javadi, A.-H., Emo, B., Howard, L.R., Zisch, F.E., Yu, Y., Knight, R., Pinelo Silva, J., and Spiers, H.J. (2017). Hippocampal and prefrontal processing of network topology to simulate the future. Nat. Commun. *8*, 14652.
