# Supplementary material for: Hippocampal place cells encode global location but not connectivity in a complex space
Source: Curr Biol. 2021 Mar 22;31(6):1221–1233.e9. doi: 10.1016/j.cub.2021.01.005 (PMC7988036; doi:10.1016/j.cub.2021.01.005)
Supplement: Document S2. Article plus supplemental information [file mmc4.pdf]

Current Biology

Article

Hippocampal place cells encode global location but not connectivity in a complex space

Highlights

- Rats flexibly navigate in a four-room maze where connectivity changes
- Place cell firing fields are not specifically altered by changes in connectivity
- Single or ensemble place cell activity does not encode changes in connectivity
- Place cells can uniquely map identical connected compartments

Authors

Éléonore Duvelle, Roddy M. Grieves, Anyi Liu, ..., Christopher Summerfield, Giovanni Pezzulo, Hugo J. Spiers

Correspondence

e.duvelle@ucl.ac.uk (É.D.), h.spiers@ucl.ac.uk (H.J.S.)

In Brief

Hippocampal place cells are thought to underlie a "cognitive map" of space. Duvelle and Grieves et al. show that the place cells of rats discriminate four connected rooms and rats learn changes in their connectivity. However, such changes do not affect place cell activity, suggesting that other brain regions or mechanisms encode this information.

Duvelle et al., 2021, Current Biology *31*, 1221–1233
March 22, 2021 © 2021 The Authors. Published by Elsevier Inc.
https://doi.org/10.1016/j.cub.2021.01.005

CellPress

Current Biology

CellPress
OPEN ACCESS

Article

Hippocampal place cells encode global location but not connectivity in a complex space

Éléonore Duvelle,[1,2,6,7,*] Roddy M. Grieves,[1,2,6] Anyi Liu,[1] Selim Jedidi-Ayoub,[1] Joanna Holeniewska,[1] Adam Harris,[3] Nils Nyberg,[1] Francesco Donnarumma,[4] Julie M. Lefort,[5] Kate J. Jeffery,[1] Christopher Summerfield,[3] Giovanni Pezzulo,[4] and Hugo J. Spiers[1,*]
[1]Department of Experimental Psychology, Institute of Behavioural Neuroscience, University College London, London, UK
[2]Department of Psychological and Brain Sciences, Dartmouth College, Hanover, NH, USA
[3]Department of Experimental Psychology, University of Oxford, OX2 6BW Oxford, UK
[4]Institute of Cognitive Sciences and Technologies, National Research Council, via S. Martino d. Battaglia 44, 00185 Rome, Italy
[5]University College London, Department of Cell and Developmental Biology, London, UK
[6]These authors contributed equally
[7]Lead contact
*Correspondence: e.duvelle@ucl.ac.uk (É.D.), h.spiers@ucl.ac.uk (H.J.S.)
https://doi.org/10.1016/j.cub.2021.01.005

SUMMARY

Flexible navigation relies on a cognitive map of space, thought to be implemented by hippocampal place cells: neurons that exhibit location-specific firing. In connected environments, optimal navigation requires keeping track of one's location and of the available connections between subspaces. We examined whether the dorsal CA1 place cells of rats encode environmental connectivity in four geometrically identical boxes arranged in a square. Rats moved between boxes by pushing saloon-type doors that could be locked in one or both directions. Although rats demonstrated knowledge of environmental connectivity, their place cells did not respond to connectivity changes, nor did they represent doorways differently from other locations. Place cells coded location in a global reference frame, with a different map for each box and minimal repetitive fields despite the repetitive geometry. These results suggest that CA1 place cells provide a spatial map that does not explicitly include connectivity.

INTRODUCTION

Real-world navigation involves traversing complex environments. How are these represented by the brain? Environmental features can be contextual (e.g., color and odor), topographic (e.g., angles and distances), or topologic (relationships preserved through deformation: containment, adjacency, connectivity). Hippocampal place cells, with their location-specific firing,[1] are thought to form the basis of a cognitive map underlying spatial memory and flexible navigation.[2] Place cells encode topographical and contextual information, specifically environment geometry,[3,4] color,[5] texture,[6] and size.[7,8] Topology could be encoded implicitly in the co-firing[9,10] or ensemble activity[11] of place cell activity. Here, we investigated whether they encode connectivity changes explicitly in their firing rate and location.

Barriers change the firing of nearby place cells.[8,12,13] Such local remapping is consistent with the predictions of the boundary vector cell (BVC) model, whereby place cells are driven by neurons sensitive to boundaries.[14,15] Another recent proposal suggests that place cells form a predictive map or "successor representation" of environments.[16,17] In this view, altering connectivity impacts future states, thus changing the firing of local firing fields ("place fields"). To our knowledge, no experiment has directly investigated the effect of connectivity changes on hippocampal activity while maintaining geometry and controlling for behavioral changes known to influence place cells such as movement direction[18,19] or running speed.[20]

To test this, we used an environment with four geometrically identical boxes connected by pushable doors. Environmental connectivity could be seamlessly modified by locking doors. We trained rats in a task alternating goal-directed navigation to a sound-cued box and pseudo-random foraging in that box. Rats demonstrated knowledge of connectivity, but the firing of their place cells did not change with connectivity. Place cells instead represented spatial location in a global reference frame, discriminating between boxes.

RESULTS

Performance in the four-room navigation task

Behavioral data were obtained from 5 rats in the "four-room" environment (see Figures 1 and S1; Video S1; STAR methods, "Training" and "Task") in two different sequences of sessions. Both started with two control sessions with all doors unlocked, followed by two test sessions where either one of the doors was locked both ways ("Closed-Door" sequence, Figure 1B) or all doors were locked one way ("One-Way" sequence) (Figure 1C), and ended with a final control session. Sessions consisted of 12 trials. For each trial, the experimenter rang a bell attached to a chosen goal box and threw food in that box until the rat finished foraging. Rats continuously alternated between foraging within a box and "goal-directed" trajectories between boxes. This allowed homogeneous spatial sampling and avoided the use of a response

This is an open access article under the CC BY license (http://creativecommons.org/licenses/by/4.0/).

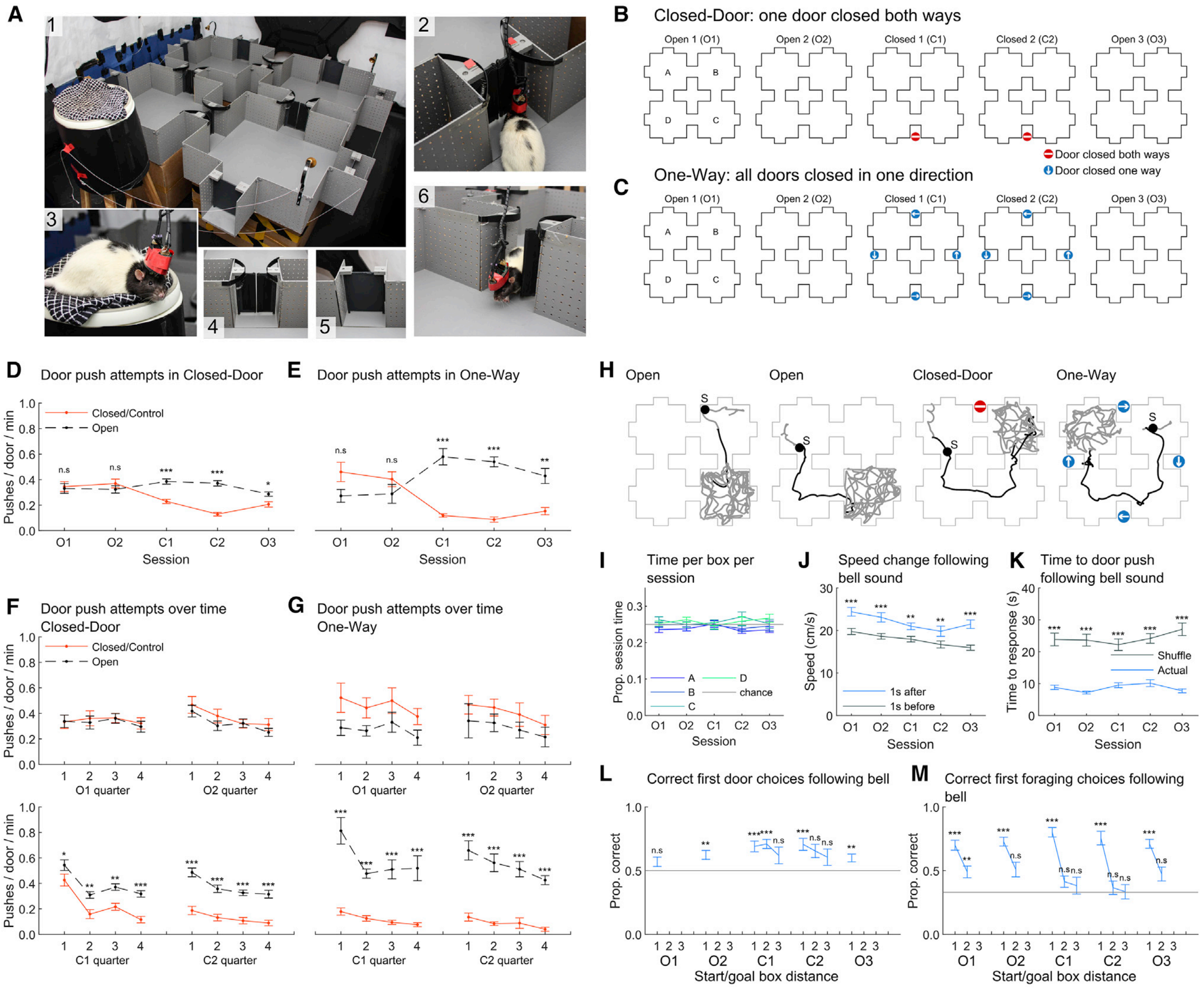

Figure 1. Protocol and behavior showing connectivity knowledge

(A) Photographs of (1) the maze, note the bells attached to each box. The same goal box order was used for all 5 sessions of a sequence. See Figure S1A. (2) Rat pushing a one-way door; (3) rat on the elevated platform, (4) normal door, (5) "dummy" door, and (6) rat passing through an open door.

(B) Closed-Door sequence protocol. Stop signs indicate locked doors.

(C) One-Way sequence protocol. Blue arrows indicate the open direction with the other direction locked.

(D and E) Normalized number of pushes on a door side for control doors (in O1–3, doors that will be or were locked), locked doors (same doors but in C1 and C2), or open doors, averaged over sessions.

(F and G) Same as (D) and (E) separated by session quarters. See also Figures S1D, S1E, and S1I.

(H) Example trials: black markers indicate rat position when the bell rang; bell symbol indicates origin of the sound. Thick gray lines show trajectories classified as foraging. Black lines show goal-directed trajectories.

For (I)–(M), Closed-Door and One-Way sequences are combined.

(I) Average time spent in each box in each session.

(J) Average movement speed 1 s before or after a bell sound.

(K) Average time between bell sound and next door push.

(L) Proportion of optimal first door pushes after a bell sound, separated by optimal distance between start and goal box (1 = adjacent boxes). Gray line indicates chance level.

(M) Proportion of correct first foraging choices. Chance level (gray) is 33%, excluding the initial box.

See Figures S1F and S1G for plots separated by sequence type. Here and later, error bars indicate standard error of the mean and *$p < 0.05$, **$p < 0.01$, ***$p < 0.001$. See also Video S1 and Figure S1.

strategy not necessarily engaging the hippocampus.[21,22] Trajectories were automatically classified (Figures 1H and S3A; STAR methods, "Behavior categorization"). Sessions lasted 23 min on average with a 10-min rest period between them. We used 19 Closed-Door sequences and 11 One-Way sequences (Table S1).

CellPress
OPEN ACCESS

Table 1. Statistics for Figure 1

Panel and dependent variable	Omnibus test	Effect of group (closed/control or open)	Effect of session (O1, O2, C1, …)	Interaction group X session	Post hoc tests across groups (corrected[a])
D, pushes/door/min	Two-way repeated-measures ANOVA	F(1, 72) = 40.6, p < 0.001	F(4, 72) = 7.6, p < 0.001	F(4, 72) = 27.9, p < 0.001	C1: p < 0.001<br>C2: p < 0.001<br>O3: p = 0.021<br>All other p > 0.75
E, pushes/door/min		F(1, 40) = 7.2, p = 0.022	F(4, 40) = 2.6, p = 0.053	F(4, 40) = 21.4, p < 0.001	C1: p < 0.001<br>C2: p < 0.001<br>O3: p < 0.006<br>all other p > 0.15
D versus E<br>Closed-Door versus One-Way difference		Group = Closed-Door versus One-Way F(1, 140) = 7.9, p = 0.0056	F(4, 140) = 32.5, p < 0.001	F(4, 140) = 7.5, p < 0.001	C1: p = 0.0016,<br>All other p > 0.13
F<br>Session quarter pushes/door/min, O1		F(1, 54) = 0.5, p = 0.493	F(3, 54) = 0.7, p = 0.576	F(3, 54) = 0.4, p = 0.772	N/A
F<br>Session quarter pushes/door/min, O2		F(1, 54) = 2.6, p = 0.126	F(3, 54) = 5.0, p = 0.004	F(3, 54) = 0.7, p = 0.553	N/A
F<br>Session quarter pushes/door/min, C1		F(1, 54) = 53.5, p < 0.001	F(3, 54) = 23.6, p < 0.001	F(3, 54) = 0.9, p = 0.434	Q1: p = 0.044<br>Q2: p = 0.003<br>Q3: p = 0.002<br>Q4: p < 0.001
F<br>Session quarter pushes/door/min, C2		F(1, 54) = 102.1, p < 0.001	F(3, 54) = 9.3, p < 0.001	F(3, 54) = 1.6, p = 0.192	All p < 0.001
G<br>Session quarter pushes/door/min, O1		F(1, 30) = 3.0, p = 0.117	F(3, 30) = 2.7, p = 0.064	F(3, 30) = 0.2, p = 0.900	N/A
G<br>Session quarter pushes/door/min, O2		F(1, 30) = 0.9, p = 0.367	F(3, 30) = 2.9, p = 0.0497	F(3, 30) = 0.0, p = 0.994	N/A
G<br>Session quarter pushes/door/min, C1		F(1, 30) = 40.4, p < 0.001	F(3, 30) = 9.0, p < 0.001	F(3, 30) = 4.2, p = 0.014	All p < 0.001
G<br>Session quarter pushes/door/min, C2		F(1, 30) = 97.4, p < 0.001	F(3, 30) = 4.8, p = 0.007	F(3, 30) = 1.1, p = 0.371	All p < 0.001
I<br>Time per box in every session		F(3, 216) = 1.2, p = 0.300	F(4, 216) = 1.2, p = 0.340	F(12, 216) = 1.0, p = 0.430	N/A
J<br>Speed change post- bell		F(1, 116) = 46.9, p < 0.001	F(4, 116) = 9.7, p < 0.001	F(4, 116) = 2.2, p = 0.072	All p < 0.001
K<br>Time to door push post-bell		F(1, 116) = 183.6, p < 0.001	F(4, 116) = 2.6, p = 0.042	F(4, 116) = 8.1, p < 0.001	All p < 0.001

(*Continued on next page*)

Table 1. *Continued*

Panel and dependent variable	Omnibus test	Effect of group (closed/control or open)	Effect of session (O1, O2, C1, …)	Interaction group X session	Post hoc tests across groups (corrected[a])
L<br>Correct first door choices post-bell	independent 1-sample t tests with Holm-Bonferroni correction	compared to chance (1/2): from left to right: p > 0.99, p = 0.003, p < 0.001, p < 0.001, p > 0.99, p > 0.99, p < 001, p > 0.99, p > 0.99, p > 0.99			
M<br>Correct first foraging post-bell		compared to chance (1/3): From left to right: p < 0.001, p = 0.002, p < 0.001, p > 0.99, p < 0.001, p > 0.99, p > 0.99, p < 0.001, p > 0.99, p > 0.99, p < 0.001, p > 0.99			

[a]Note: post hoc tests were based on estimated marginal means and corrected using the Tukey-Kramer method.

Rats rapidly learned spatial connectivity changes

In both sequences, rats pushed on all doors equally in the first (O1) and second (O2) control sessions but significantly less on locked doors in the third and fourth sessions (C1 and C2) (STAR methods, "Event flags"; Table 1 for statistics). Although all doors reopened in the 5th session (O3), rats still pushed less on the previously locked doors, indicating long-term memory for door state and an incomplete updating of connectivity; for this reason, our analyses do not generally rely on session O3. These effects hold across individual rats (Figure S1E). Rats pushed significantly less on locked door sides in the C1 session in One-Way sequences compared to Closed-Door sequences, suggesting better knowledge of connectivity in One-Way sequences. Rats discriminated between locked and open doors from the first time quarter of sessions, indicating fast learning (Figures 1F, 1G, and S1D).

Rats associated the bell with a change in goal

We next assessed rats' knowledge of the task; see Figure 1H for representative trial trajectories. Session duration and time per box did not significantly vary with session ($p > 0.05$ in all cases, univariate ANOVA) (Figure 1I; Table 1 for all statistics). Rats ran faster and were more likely to push on a door after a bell sound (Figures 1J and 1K). The first door that rats interacted with following a bell sound tended to be the optimal one, but this was not statistically significant for all conditions (Figure 1L). The first box rats foraged in following a bell sound was the correct one only when adjacent to the start (Figure 1M), suggesting that rats often foraged in boxes on the way to the goal. Data separated by sequence type are shown in Figures S1F and S1G. In summary, rats seemed aware that the bell sound indicated a new goal and usually, but not always, chose the optimal path to it.

Place cell activity in the four-room environment

We recorded CA1 extracellular activity from the same 5 rats and 30 sequences of sessions. Only putative CA1 place cells were analyzed (STAR methods, "Unit classification," "Firing rate maps and spatial information," and "Histology;" Figures S1B and S1C). We recorded 261 place cells in Closed-Door (out of 325 neurons, 80.3%) and 161 place cells in One-Way (out of 232 neurons, 69.4%) sequences; these proportions were not statistically different ($\chi^2(1) = 0.021$, $p = 0.89$, chi-square test of expected proportions). Tables S1 and S2 and Figure S2 show sequence and cell counts, cluster quality measures, and example cells, respectively.

Place cells did not respond to spatial connectivity changes

Our main hypothesis was that CA1 place cells, as the neural substrate of a cognitive map, explicitly encode environmental connectivity. As behavior during goal-directed transitions would strongly differ depending on connectivity, which might spuriously influence place cell firing, we analyzed only foraging periods unless otherwise specified (see Figure S1H). Although the firing rate of cells generally increased in the goal-directed phase (Figure S3C) this was likely linked to an increase in movement speed (Figure S3B). There was no global spatial remapping between the two task phases (Figures S3D and S3E).

We first asked whether place cells globally encoded connectivity status by comparing firing rate maps between sessions (Figures 2B and 2H; STAR methods, "Rate map correlations;" Table 2 for statistical results). Correlations between consecutive sessions were high and correlations between sessions O2-C1, where connectivity changed, were not different from O1-O2, which had identical connectivity (Figures 2C and 2I). Instead, correlations significantly decreased with time (Figures 2D and 2J), coherent with past findings.[23] We next focused on activity local to the changes (25 cm radius around doors, Figures 2A and 2G). There were no significant differences between O1-O2 and O2-C1 correlations, either for Closed-Door (Figure 2E) or One-Way (Figure 2K) sequences. We also examined rate

CellPress
OPEN ACCESS

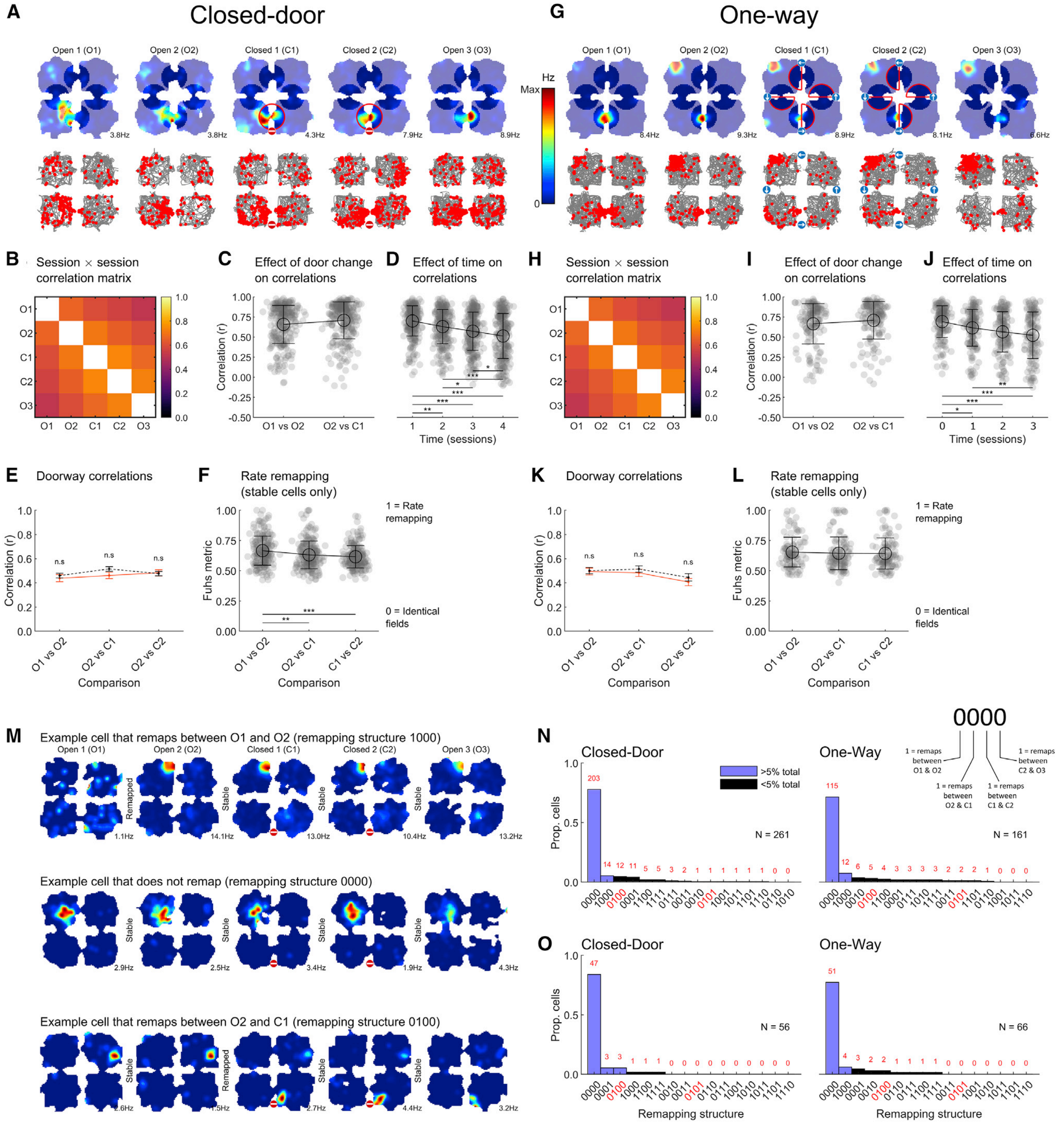

Figure 2. Place cell firing was not altered by connectivity changes

Analyses exclude goal-directed epochs. See Figures S4A–S4L for analysis of pyramidal non-place cells.

(A) Example cell from Closed-Door sequences. (Top) Foraging rate maps, colors indicate firing rate, number indicates maximum rate, non-shaded areas are used in the doorway correlation analysis in (E), and red circled areas correspond to the locked door. (Bottom) Foraging spike plots, gray: trajectory, red: spikes. See also Figure S2.

(B) Matrix of cell-averaged rate map correlations between all sessions.

(C) Comparison of correlations between two open sessions (O1&O2) and one open and one locked (O2&C1).

(D) Correlations between rate maps as a function of number of sessions between them, 0 = consecutive sessions (O1&O2, O2&C1, etc.), 1 = pairs with 1 session between them (O1&C1, C1&O3, etc.), etc.

(E) Correlations focusing on 25 cm around doors.

(legend continued on next page)

Table 2. Statistics for Figure 2

Panel and dependent variable	Omnibus test	Effect of group (closed/control or open)	Effect of session	Interaction group × session	Post hoc tests across groups (corrected[a])
C<br>Correlations<br>O1-O2 versus O2-C1	one-way ANOVA	N/A	F(1, 509) = 6.0, p = 0.015	N/A	N/A
D<br>Correlations over sessions/time	one-way ANOVA	N/A	F(3, 1017) = 30.4, p < 0.001	N/A	1v2: p = 0.003 1v3: p < 0.001 1v4: p < 0.001 2v3: p = 0.030 2v4: p < 0.001 3v4: p = 0.020
E<br>Doorway correlations	two-way ANOVA	F(1, 1238) = 1.6, p = 0.212	F(2, 1238) = 1.7, p = 0.190	F(2, 1238) = 0.9, p = 0.400	N/A
F<br>Fuhs rate remapping metric	one-way ANOVA	N/A	F(2, 598) = 11.0, p < 0.001	N/A	O1vsO2 versus O2vsC1: p = 0.004 O1vsO2 versus C1vsC2: p < 0.001 All other p > 0.30
I<br>Correlations<br>O1-O2 versus O2-C1	one-way ANOVA	N/A	F(1, 509) = 6.0, p = 0.015	N/A	N/A
J<br>Correlations over sessions/time	one-way ANOVA	N/A	F(3, 602) = 13.8, p < 0.001	N/A	1v2: p = 0.022 1v3: p < 0.001 1v4: p < 0.001 2v4: p = 0.005 All other p > 0.25
K<br>Doorway correlations	two-way ANOVA	F(1, 774) = 1.2, p = 0.274	F(2, 774) = 4.2, p = 0.015	F(2, 774) = 0.2, p = 0.849	N/A
L<br>Fuhs rate remapping metric	one-way ANOVA	N/A	F(2, 330) = 0.2, p = 0.78	N/A	N/A

[a]Note: all post hoc tests were based on estimated marginal means and corrected using the Tukey-Kramer method.

remapping[3] by using a normalized index that equals 0 for identical fields and approaches 1 when the firing rate differs[24] (STAR methods, "Firing rate remapping"). Rate remapping did not increase when comparing O2-C1 to O1-O2 (Figures 2F and 2L), indicating that the population of place cells did not encode a change of connectivity in their firing rates. Similar results were found when analyzing pyramidal non-place cells, with the exception of the time effect (Figures S4A–S4L).

Individual place cells did not remap with connectivity changes

We then computed remapping indices for individual cells by comparing the correlations between consecutive sessions to a shuffle (Figure 2M; STAR methods, "Individual remapping between sessions"). In Closed-Door sequences, only 13 place cells (4.5%) had a pattern indicative of connectivity remapping (Figure 2N). Because these numbers were equivalent to or lower than those of cells remapping between O1 and O2, they do not appear to reflect any connectivity-specific effect. Most cells were significantly more stable than chance (203, 77.8%). Similar results were found for cells with a field near locked doors (Figure 2O) with 3 out of 56 (5.4%) connectivity-remapping cells. Similar results were obtained for One-Way (Figures 2N and 2O) sequences.

Spatial connectivity changes did not alter place field properties

We next analyzed individual place fields (STAR methods, "Place fields"). All field centroids are shown in Figures 3A and 3E. The

(F) The rate remapping index significantly decreased for the comparisons with the connectivity change indicating less, not more, remapping when the connectivity was modified.
(G–L) Same presentation as (A)–(F) for One-Way sequences, with similar results.
(M) Example foraging rate maps.
(N) Histograms showing remapping patterns for all place cells. Red axis labels highlight conditions where remapping could be attributed to a change of connectivity.
(O) Similar to (N) but only for cells with a field within 25 cm of the locked door(s).
See also Video S2 for activity plots of all used place cells, Figures S2–S4.

CellPress
OPEN ACCESS

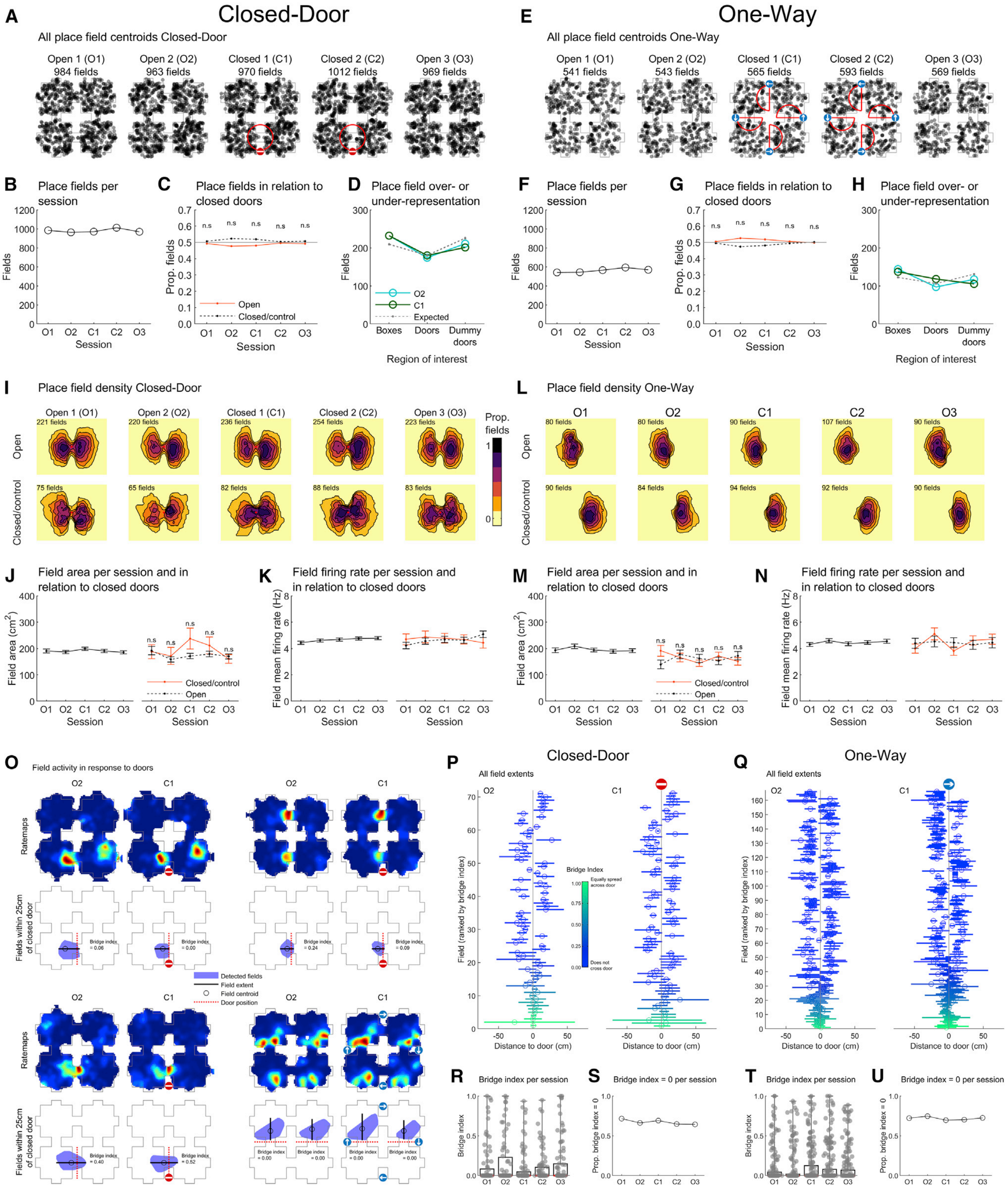

Figure 3. No door overrepresentation and no change in place field properties around locked doors

(A)–(N) use foraging epochs only, while (O)–(U) also uses goal-directed epochs. See also Figure S5.

(A) Spatial distribution of all place field centroids for Closed-Door sequences centered on door location.

(B) Number of place fields in each session.

(C) Proportion of fields within 25 cm of a door.

(legend continued on next page)

number of fields active (Figures 3B and 3F) or close to locked doors (Figures 3C and 3G) did not change across sessions (Table 3 for all statistics). Place fields might over-represent doors, as reported previously for entryways into compartments.[4,25] We compared the density of place fields around doors, peripheral "dummy" doors, and inside boxes to numbers expected from a uniform distribution and found that place fields did not significantly overrepresent any of these locations (Figures 3D and 3H; STAR methods, "Place field overrepresentation").

Place field size (STAR methods, "Place field firing rate and area") also did not significantly change across sessions and fields next to locked doors did not change in size more than those next to unchanged doors (Figures 3I, 3J, 3L, and 3M). Similarly, there was no difference in local firing rate (Figures 3K and 3N). We also investigated whether place fields moved in response to a locked door by tracking absolute centroid shift across sessions. Fields shifted more between O1 versus O2 than between O2 and C1, indicating that connectivity changes do not specifically induce field displacement (see STAR methods, "Field distance to doorway" and Figure S5).

We next asked whether fields extent across doors changed with connectivity (STAR methods, "Place field extent across doors"). Focusing only on local fields (centroid in a 25 cm radius from the changing door), we computed a "bridge index" as the difference between the area on each side of a door normalized by the total area (Figure 3O). This index ranges from 0 (no crossing) to 1 (equal split). For visualization, we extracted the length of the field extent on the axis orthogonal to the door axis. Goal-directed data were included to ensure that fields would not be artificially split. We first found that most place fields did not extend through doors, as indicated by a majority of zero values (Figures 3P and 3Q). There were no significant differences in bridge index between sessions (Closed-Door: $\chi^2(4,406) = 1.6$, $p > 0.80$; One-Way: $\chi^2(4,917) = 1.7$, $p > 0.70$; Kruskal-Wallis tests) (Figures 3R and 3T). The proportion of fields exhibiting a bridge index of zero in O2 and C1 also did not differ significantly (Closed-Door: $z = -0.39$, $p = 0.70$; One-Way: $z = 1.06$, $p = 0.29$, Z-score tests of two population proportions) (Figures 3S and 3U). Finally, an analysis of average place cell firing when entering a box found no effect of the number of available connections in that box (Figures S4M and S4N).

Place cells encoded position mainly in a global reference frame

In previous experiments using four connected compartments with identical orientation and geometry, most place cells fired in the same place in each compartment,[4,25,26] and rats were impaired in a spatial task.[4] To assess how place cells encode the four-room environment, we first quantified the number of place fields per cell: more than 50% of cells had 3 or less detected place fields (Figure 4A) suggesting that place field repetition was uncommon. We computed the average correlation between maps for every box (Figure 4B). Diagonal bands of high correlations indicated that box representations were highly self-similar across sessions but not between different boxes (self versus other boxes, Closed-Door: $t(182) = 58.7$, $p < 0.001$; One-Way: $t(182) = 58.4$, $p < 0.001$, two-sample t tests). Correlations were also not higher for adjacent boxes than diagonally opposite ones (Closed-Door (O2): $F(1, 506) = 0.1$, $p = 0.78$; Closed-Door (C1): $F(1,502) = 0$, $p = 0.84$; One-Way (O2): $F(1,290) = 0$, $p = 0.93$; One-Way (C1): $F(1,301) = 0.6$, $p = 0.46$, One-Way ANOVAs) (Figure 4C; STAR methods, "Firing rate maps and spatial information").

We next focused on the distribution of cross-box correlations for O2 only (Figure 4D, blue distribution). It was skewed toward low values (median 0.10) in contrast to experiments reporting place field repetition (median correlations >0.5).[4,25,26] We designed shuffles to simulate cross-box correlations expected under different outcomes: non-repeating or repeating fields in 2, 3, or all 4 compartments (STAR methods, "Place field repetition"). Data differed significantly from all shuffle distributions except for 2 repeating fields (Figure 4D) ($p > 0.05$, $p < 0.001$ in all other cases, two-sample Kolmogorov-Smirnov tests, Holm-Bonferroni corrected), indicating a small but higher than chance amount of field repetition. When cells were classified by place field number, all correlation distributions with more than one field presented a bimodality, revealing a small subpopulation of cells with high (>0.5) correlation values indicative of repeating fields (Figure 4E; see Figures S6A and S6B for One-Way sequences). Thus, place field repetition is on a continuum: although most place cells did not repeat, some did in varying subsets of the boxes. Cells exhibiting repeating fields could be co-recorded with non-repeating ones, and some cells expressed repeating as well as non-repeating fields (Figures 4D and 4E). This pattern did not change with connectivity (Figures S6C and S6D).

To further assess whether place cells implemented a global coding of space, we decoded box quadrants from firing activity using Bayesian decoding[27,28] (STAR methods, "Bayesian decoding of box quadrants") (Figure 4F). This analysis used both foraging and goal-directed data. To avoid overfitting,[29] we

(D) Number of detected or expected fields around doors or dummy doors or inside a box.
(E–H) Same as (A)–(D) but for One-Way sequences.
(I) Contour maps showing proportion of all fields active in each pixel around doors.
(J) Field area either for all data (left) or only next to a door (right).
(K) Mean infield firing rate across sessions for all data (left) or only door place fields (right).
(L–N) Same as (I)–(K) but for One-Way sequences.
(O) Example rate maps (O2 and C1) from One-Way (bottom right) and Closed-Door (others) sequences with a field around a changed door—only such fields were analyzed. Below are maze schematics with fields represented by a blue polygon and doors as a red line. Text gives the bridge index of each field.
(P) For visualization, all door fields from the Closed-Door sequence. Lines show field extent along the axis perpendicular to the door; circles give field centroid along this axis. Fields are sorted from bottom to top from high to low bridge index.
(R) Closed-Door bridge indices in all sessions.
(S) Proportion of fields with a bridge index of 0 in each session.
(Q, T, and U) Same as (P), (R), and (S) for One-Way sequences. The arrow indicates the open direction.
See also Figure S5.

Table 3. Statistics for Figure 3

Panel and dependent variable	Omnibus test	Result (B–H) or effect of session (J–N)	Effect of group (J–N)	Post Hoc tests across groups (corrected[a], B–H) or interaction group × session (J–N)
B<br>Fields per session, Closed-Door	chi-square test of equal prop	$X^2(4, n= 4,898) = 1.58$, $p = 0.810$	N/A	N/A
C<br>Fields closed/open boxes Closed-Door	chi-square test of equal prop (chance 50%)	N/A	All $p > 0.99$	All $p > 0.99$
D<br>Place field over- or under-representation, Closed-Door O2	chi-square test of equal prop (compared to expected)	$X^2(2, n = 951) = 3.59$, $p = 0.170$	All $p > 0.99$	All $p > 0.99$
D<br>Place field over- or under-representation, Closed-Door C1	chi-square test of equal prop (compared to expected)	$X^2(2, n = 951) = 5.14$, $p = 0.077$	N/A	N/A
F<br>Fields per session protocol One-Way	chi-square test of equal prop	$X^2(4, n = 2,811) = 3.24$, $p = 0.520$	N/A	N/A
G<br>Fields closed/open boxes One-Way	chi-square test of equal prop (chance 50%)	N/A	All $p > 0.99$	All $p > 0.99$
H<br>Place field over- or under-representation, One-Way O2	chi-square test of equal prop (compared to expected)	$X^2(2, n = 560) = 6.10$, $p = 0.047$	All $p > 0.99$	All $p > 0.99$
H<br>Place field over- or under-representation, One-Way C1	chi-square test of equal prop (compared to expected)	$X^2(2, n = 560) = 8.19$, $p = 0.017$	All $p > 0.99$	All $p > 0.99$
J left<br>Field area between sessions, Closed-Door	one-way ANOVA	$F(4, 4893) = 0.7$, $p = 0.590$	N/A	N/A
J right<br>Field area per session per doorway, Closed-Door	two-way ANOVA	$F(4, 1537) = 1.8$, $p = 0.120$	$F(1, 1537) = 2.8$, $p = 0.100$	$F(4, 1537) = 1.4$, $p = 0.230$
K left<br>Field frate between sessions, Closed-Door	one-way ANOVA	$F(4, 4893) = 1.4$, $p = 0.227$	N/A	N/A
K right<br>Field frate per session per doorway, Closed-Door	two-way ANOVA	$F(4, 1537) = 0.3$, $p = 0.910$	$F(1, 1537) = 0.1$, $p = 0.770$	$F(4, 1537) = 0.8$, $p = 0.510$
M left<br>Field area between sessions, One-Way	one-way ANOVA	$F(4, 2806) = 0.9$, $p = 0.470$	N/A	N/A
M right<br>Field area per session per doorway, One-Way	two-way ANOVA	$F(4, 887) = 0.4$, $p = 0.840$	$F(1, 887) = 0.1$, $p = 0.710$	$F(4, 887) = 1.9$, $p = 0.110$
N left<br>Field frate between sessions, One-Way	one-way ANOVA	$F(4, 2806) = 0.7$, $p = 0.610$	N/A	N/A
N right<br>Field frate per session per doorway, One-Way	two-way ANOVA	$F(4, 887) = 1.1$, $p = 0.360$	$F(1, 887) = 0.0$, $p = 0.900$	$F(4, 887) = 0.9$, $p = 0.440$

[a]Post hoc tests were individual chi-square tests of equal proportions, and p values were corrected using the Holm-Bonferroni method (rejected results are represented as $p > 0.99$, remaining values are not adjusted).

used maps from O1 (or O2) to decode the instantaneous firing in O2 (or C1, respectively). In all "confusion matrices," we observed a clear diagonal (Figure 4G), indicating accurate decoding to the correct box and quadrant. The average decoding values on the diagonal were above chance, whereas other categories (decoding of correct quadrant in wrong box, or incorrect quadrant in correct box) were not (Figure 4H). We also compared the probability of correct decoding between O1-O2 and O2-C1 conditions, for Closed-Door sequences: if the activity changed with connectivity, decoding performance would drop when using O2 maps to decode C1 data (Figure 4H); instead, we found no significant difference ($t(8) = -1.60$, $p = 0.147$, paired t test). No

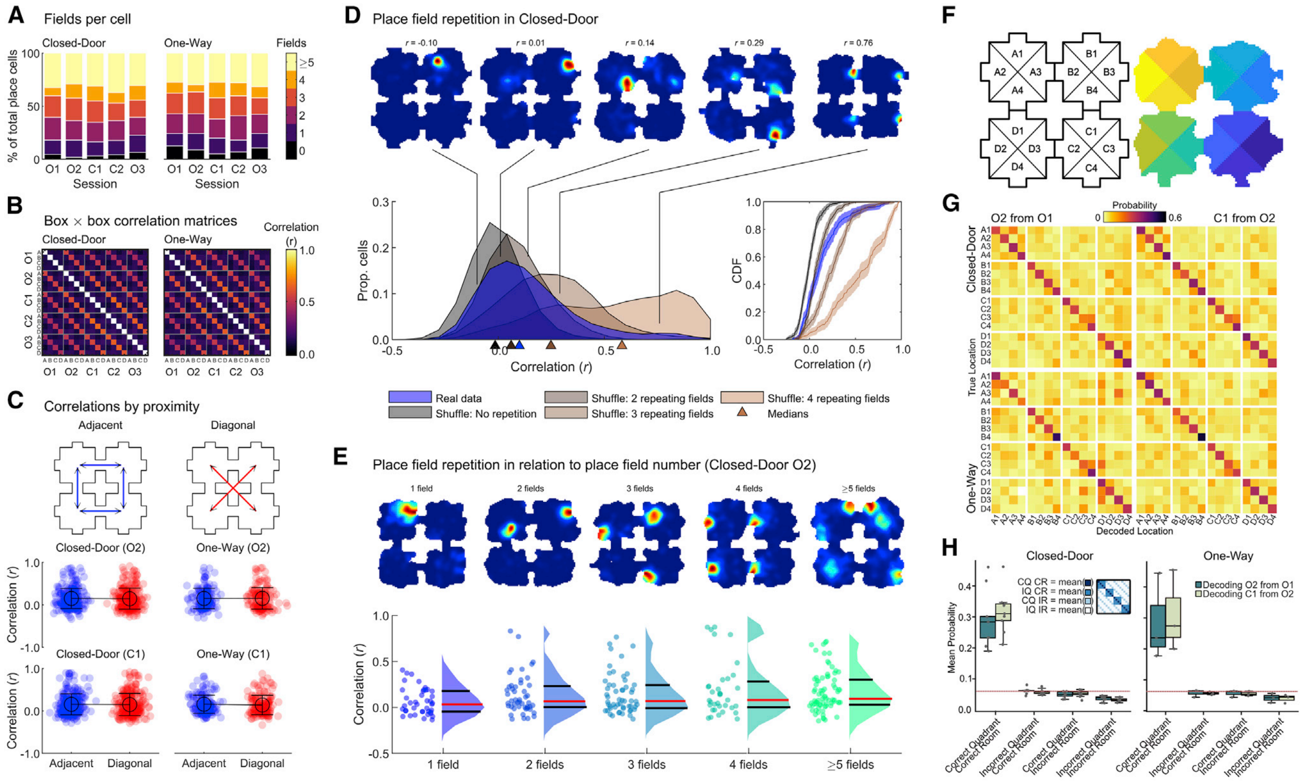

Figure 4. Most place cells encode global position in the four-room maze

(A) Percentage of place cells grouped by number of detected place fields.

(B) Cross-box correlation matrices averaged over all place cells.

(C) Paired rate map correlations between adjacent or diagonal boxes.

(D) (D) and (E) show data for O2, Closed-Door sequences. Shown on top, example rate maps (all data) with corresponding correlation values from the data distribution (blue). On the bottom left, the blue distribution represents cross-box correlation values for session O2. Other distributions represent shuffles simulating the correlations expected for different field repetitions. x axis markers indicate the distribution medians. On the bottom right, the same data is presented as empirical cumulative distribution functions; shaded areas represent lower and upper confidence intervals.

(E) Top, example rate maps for each place field category. Shown on the bottom, cross-box correlations scores for all data, separated by number of fields; dots correspond to individual place cells, and data distributions are shown as violin plots with median in red and quartiles in black.

(F) Box quadrants used for Bayesian decoding with example binned position data.

(G) Mean confusion matrices from sequences with at least 15 simultaneously recorded place cells from Closed-Door (n = 9) and One-Way (n = 3) sequences; see Figure S7.

(H) Average decoding performance for different categories (see inset). Points indicate session-average probabilities per quadrant for each category. Red line indicates chance performance (1/16 quadrants).

See also Figures S6 and S7.

test was run on One-Way sequences given the small sample size (3 sessions), but the distributions appear similar.

In summary, the population of place cells represented position with a mostly different map for each room, and this was independent of connectivity status.

DISCUSSION

We tested whether hippocampal CA1 place cells encode changes in the connectivity of a four-room environment in their firing rate patterns. Although we found that rats rapidly learned connectivity changes and place cells accurately encoded global location (intermixed with a subset showing local coding), we found no evidence for connectivity encoding. Our results clarify the extent to which CA1 contributes to representing connected environments, which will aid the refinement of models of hippocampal mechanisms.

Navigation in connected spaces

Rats and humans can flexibly adapt to changes in environmental connectivity.[13,30–39] Here, rats adapted to the locked or unlocked status of doors, even door sides, in the absence of directly perceivable changes. Importantly, their bias toward open doors persisted into the last session suggesting learning of connectivity.[13] The observation of direct, detour-like trajectories around locked doors suggest rats were using a map-based strategy,[2,40] but future experiments would help assess this as trajectories were not always significantly optimal, which could be due to the low cost of foraging.

Place cells do not explicitly encode spatial connectivity

Environmental connectivity has long been seen as an integral part of the cognitive map.[30,41,42] Here, a very small proportion of place cells remapped when connectivity changed, not more than when

it was unchanged, suggesting non-specificity to connectivity updating but perhaps latent time coding.[23,43] We did not detect any effects of changing the connectivity in spatial correlations, firing rates, field locations, field sizes, or field extent through doorways, even on activity local to the changed doors. Doors were not overrepresented, in contrast to previous studies with open doorways.[25,44] Thus, after controlling for confounding factors, such as occupancy, speed, and movement direction (by analyzing comparable behavior), time (by comparing pairs of sessions separated by a similar time difference), and geometry (by locking existing doors instead of introducing a new barrier), we found no evidence of explicit encoding of connectivity by CA1 place cells.

In contrast, a past study[13] found local remapping after introducing a barrier creating a detour; however, this experimental manipulation also changed the rats' behavior and environmental geometry, both of which could have impacted place cells.[12,14,15] Opening a path might be encoded differently from closing one as CA3 remapping happened far from a new shortcut,[32] which we could not test in this experiment. Thus, environmental connectivity could be encoded in CA3, in the subiculum[45,46] or the medial entorhinal cortex.[47,48] The prefrontal cortex could be involved, as its activity levels vary depending on available spatial connections in humans.[49,50] Finally, connectivity could be encoded implicitly either in the co-firing of place cells[9] or in non-local population phenomena such as place cell "replay"[11,51,52]; this could be examined in future experiments simultaneously recording many cells.[53]

Implications for models of place cell dynamics

The successor representation (SR) model proposes that place cells instantiate a predictive map, representing each state, or position, in terms of its successor states, or possible future positions.[16,54] It predicts changes in place cell firing when transitions become blocked (Figures 2, 3, and S3 in Stachenfeld et al.[16]) and reproduces experimental findings happening on a timescale similar to our study.[13] However, we found no evidence of such changes. The patterns of place cell dynamics best explained by the SR model might be observed under repetitive behaviors linked to more consistent policies.

The BVC model explains place field repetition and overrepresentation of doorways in geometrically identical environments.[14,55] We did not find this; however, it is currently unknown how biological BVCs respond to doors. A variant, the contextual gating model,[56,57] where different BVCs drive place cells in different contexts, could predict different maps for each box. Additional factors might explain box differentiation: the task, the greater salience of distal landmarks or the presence of uncontrolled local cues (self-deposited odors, rats visually differentiating real and dummy doors, etc.). However, presence of distal cues does not prevent place field repetition,[4,58–60] and rats could not differentiate compartments in a spatial discrimination task.[4] Finally, the different orientations of box entry points might have helped box disambiguation or pattern separation.[61] Indeed, the point of entry into a compartment affects place cell firing,[62,63] and in experiments evidencing place field repetition, each doorway had the same orientation.[4,24,25,26]

STAR★METHODS

Detailed methods are provided in the online version of this paper and include the following:

- KEY RESOURCES TABLE
- RESOURCE AVAILABILITY
  - Lead contact
  - Materials availability
  - Data and code availability
- EXPERIMENTAL MODEL AND SUBJECT DETAILS
- METHOD DETAILS
  - Overall summary
  - Room and experimental environment
  - Task
  - Training
  - Familiarisation
  - Door training
  - Task learning
  - Microdrive and implantation surgery
  - Screening and recording
  - Histology
  - Position tracking
  - Event flags
  - Behavior discrimination
  - Response to bell sounds
  - Correct door pushes and correct foraging
  - Neural activity analyses
  - Firing rate maps and spatial information
  - Unit classification
  - Rate map correlations
  - Individual remapping between sessions
  - Foraging versus goal-directed remapping
  - Firing rate remapping
  - Place fields
  - Place field repetition
  - Place field overrepresentation
  - Place field firing rate and area
  - Place field extent across doors
  - Bayesian decoding of box quadrants
  - Field distance to doorway
  - Cluster quality measures
- QUANTIFICATION AND STATISTICAL ANALYSIS

SUPPLEMENTAL INFORMATION

Supplemental Information can be found online at https://doi.org/10.1016/j.cub.2021.01.005.

ACKNOWLEDGMENTS

We thank Marta Huelin for providing an experimental subject and members of the Institute of Behavioural Neuroscience (UCL) and of the Department of Psychological and Brain Sciences (Dartmouth College) for support and discussions. This work was supported by a grant to H.J.S., C.S., and G.P. from the European Union's Horizon 2020 Framework Programme for Research and Innovation under the Specific Grant Agreements 785907 and 945539 (Human Brain Project SGA2 and SGA3), a grant to K.J.J. from the Wellcome Trust (WT103896AIA), and a grant to G.P. from the European Research Council (Grant Agreement 820213, ThinkAhead).

AUTHOR CONTRIBUTIONS

Project Administration, E.D.; Supervision, E.D. and H.J.S.; Writing – Original Draft Preparation, E.D., R.M.G., and A.L.; Writing – Review & Editing, E.D., R.M.G., A.L., A.H., J.M.L., K.J.J., C.S., G.P., and H.J.S.; Investigation, E.D., A.L., S.J.-A., J.H., and N.N.; Methodology, E.D., A.L., J.M.L., K.J.J., C.S.,

G.P., and H.J.S.; Conceptualization, E.D., C.S., G.P., and H.J.S.; Visualization, R.M.G., A.H., and A.L.; Data Curation, R.M.G.; Resources, R.M.G., and K.J.J.; Formal Analysis, R.M.G., A.H., and F.D.; Software, R.M.G.; Funding Acquisition, K.J.J., C.S., G.P., and H.J.S.

DECLARATION OF INTERESTS

K.J.J. is a non-shareholding director of Axona, Ltd.

Received: November 13, 2020
Revised: December 22, 2020
Accepted: January 5, 2021
Published: February 12, 2021

REFERENCES

1. O'Keefe, J., and Dostrovsky, J. (1971). The hippocampus as a spatial map. Preliminary evidence from unit activity in the freely-moving rat. Brain Res. *34*, 171–175.
2. O'Keefe, J., and Nadel, L. (1978). The Hippocampus as a Cognitive Map (Clarendon Press Oxford).
3. Leutgeb, S., Leutgeb, J.K., Barnes, C.A., Moser, E.I., McNaughton, B.L., and Moser, M.-B. (2005). Independent codes for spatial and episodic memory in hippocampal neuronal ensembles. Science *309*, 619–623.
4. Grieves, R.M., Jenkins, B.W., Harland, B.C., Wood, E.R., and Dudchenko, P.A. (2016). Place field repetition and spatial learning in a multicompartment environment. Hippocampus *26*, 118–134.
5. Jeffery, K.J., and Anderson, M.I. (2003). Dissociation of the geometric and contextual influences on place cells. Hippocampus *13*, 868–872.
6. Wang, C.-H., Monaco, J.D., and Knierim, J.J. (2020). Hippocampal Place Cells Encode Local Surface-Texture Boundaries. Curr. Biol. *30*, 1397–1409.e7.
7. Fenton, A.A., Kao, H.-Y., Neymotin, S.A., Olypher, A., Vayntrub, Y., Lytton, W.W., and Ludvig, N. (2008). Unmasking the CA1 ensemble place code by exposures to small and large environments: more place cells and multiple, irregularly arranged, and expanded place fields in the larger space. J. Neurosci. *28*, 11250–11262.
8. Muller, R.U., and Kubie, J.L. (1987). The effects of changes in the environment on the spatial firing of hippocampal complex-spike cells. J. Neurosci. *7*, 1951–1968.
9. Dabaghian, Y., Brandt, V.L., and Frank, L.M. (2014). Reconceiving the hippocampal map as a topological template. eLife *3*, e03476.
10. Chen, Z., Gomperts, S.N., Yamamoto, J., and Wilson, M.A. (2014). Neural representation of spatial topology in the rodent hippocampus. Neural Comput. *26*, 1–39.
11. Wu, X., and Foster, D.J. (2014). Hippocampal replay captures the unique topological structure of a novel environment. J. Neurosci. *34*, 6459–6469.
12. Rivard, B., Li, Y., Lenck-Santini, P.-P., Poucet, B., and Muller, R.U. (2004). Representation of objects in space by two classes of hippocampal pyramidal cells. J. Gen. Physiol. *124*, 9–25.
13. Alvernhe, A., Save, E., and Poucet, B. (2011). Local remapping of place cell firing in the Tolman detour task. Eur. J. Neurosci. *33*, 1696–1705.
14. Barry, C., Lever, C., Hayman, R., Hartley, T., Burton, S., O'Keefe, J., Jeffery, K., and Burgess, N. (2006). The boundary vector cell model of place cell firing and spatial memory. Rev. Neurosci. *17*, 71–97.
15. Hartley, T., Burgess, N., Lever, C., Cacucci, F., and O'Keefe, J. (2000). Modeling place fields in terms of the cortical inputs to the hippocampus. Hippocampus *10*, 369–379.
16. Stachenfeld, K.L., Botvinick, M.M., and Gershman, S.J. (2017). The hippocampus as a predictive map. Nat. Neurosci. *20*, 1643–1653.
17. Gustafson, N.J., and Daw, N.D. (2011). Grid cells, place cells, and geodesic generalization for spatial reinforcement learning. PLoS Comput. Biol. *7*, e1002235.
18. Muller, R.U., Bostock, E., Taube, J.S., and Kubie, J.L. (1994). On the directional firing properties of hippocampal place cells. J. Neurosci. *14*, 7235–7251.
19. Navratilova, Z., Hoang, L.T., Schwindel, C.D., Tatsuno, M., and McNaughton, B.L. (2012). Experience-dependent firing rate remapping generates directional selectivity in hippocampal place cells. Front. Neural Circuits *6*. Published online February 21, 2012. https://doi.org/10.3389/fncir.2012.00006.
20. McNaughton, B.L., Barnes, C.A., and O'Keefe, J. (1983). The contributions of position, direction, and velocity to single unit activity in the hippocampus of freely-moving rats. Exp. Brain Res. *52*, 41–49.
21. Packard, M.G., and McGaugh, J.L. (1996). Inactivation of hippocampus or caudate nucleus with lidocaine differentially affects expression of place and response learning. Neurobiol. Learn. Mem. *65*, 65–72.
22. Gasser, J., Pereira de Vasconcelos, A., Cosquer, B., Boutillier, A.-L., and Cassel, J.-C. (2020). Shifting between response and place strategies in maze navigation: Effects of training, cue availability and functional inactivation of striatum or hippocampus in rats. Neurobiol. Learn. Mem. *167*, 107131.
23. Mankin, E.A., Diehl, G.W., Sparks, F.T., Leutgeb, S., and Leutgeb, J.K. (2015). Hippocampal CA2 activity patterns change over time to a larger extent than between spatial contexts. Neuron *85*, 190–201.
24. Fuhs, M.C., Vanrhoads, S.R., Casale, A.E., McNaughton, B., and Touretzky, D.S. (2005). Influence of path integration versus environmental orientation on place cell remapping between visually identical environments. J. Neurophysiol. *94*, 2603–2616.
25. Spiers, H.J., Hayman, R.M.A., Jovalekic, A., Marozzi, E., and Jeffery, K.J. (2015). Place field repetition and purely local remapping in a multicompartment environment. Cereb. Cortex *25*, 10–25.
26. Harland, B., Grieves, R.M., Bett, D., Stentiford, R., Wood, E.R., and Dudchenko, P.A. (2017). Lesions of the Head Direction Cell System Increase Hippocampal Place Field Repetition. Curr. Biol. *27*, 2706–2712.e2.
27. Kaefer, K., Nardin, M., Blahna, K., and Csicsvari, J. (2020). Replay of Behavioral Sequences in the Medial Prefrontal Cortex during Rule Switching. Neuron *106*, 154–165.e6.
28. Zhang, K., Ginzburg, I., McNaughton, B.L., and Sejnowski, T.J. (1998). Interpreting neuronal population activity by reconstruction: unified framework with application to hippocampal place cells. J. Neurophysiol. *79*, 1017–1044.
29. van der Meer, M.A.A., Carey, A.A., and Tanaka, Y. (2017). Optimizing for generalization in the decoding of internally generated activity in the hippocampus. Hippocampus *27*, 580–595.
30. Tolman, E.C. (1948). Cognitive maps in rats and men. Psychol. Rev. *55*, 189–208.
31. Poucet, B., and Herrmann, T. (2001). Exploratory patterns of rats on a complex maze provide evidence for topological coding. Behav. Processes *53*, 155–162.
32. Alvernhe, A., Van Cauter, T., Save, E., and Poucet, B. (2008). Different CA1 and CA3 representations of novel routes in a shortcut situation. J. Neurosci. *28*, 7324–7333.
33. Alvernhe, A., Sargolini, F., and Poucet, B. (2012). Rats build and update topological representations through exploration. Anim. Cogn. *15*, 359–368.
34. Grieves, R.M., and Dudchenko, P.A. (2013). Cognitive maps and spatial inference in animals: Rats fail to take a novel shortcut, but can take a previously experienced one. Learn. Motiv. *44*, 81–92.
35. Javadi, A.-H., Patai, E.Z., Marin-Garcia, E., Margolis, A., Tan, H.M., Kumaran, D., Nardini, M., Penny, W., Duzel, E., Dayan, P., and Spiers, H.J. (2019). Prefrontal Dynamics Associated with Efficient Detours and Shortcuts: A Combined Functional Magnetic Resonance Imaging and Magnetoencenphalography Study. J. Cogn. Neurosci. *31*, 1227–1247.
36. Javadi, A.-H., Patai, E.Z., Marin-Garcia, E., Margois, A., Tan, H.M., Kumaran, D., Nardini, M., Penny, W., Duzel, E., Dayan, P., and Spiers, H.J. (2019). Backtracking during navigation is correlated with enhanced

CellPress
OPEN ACCESS

anterior cingulate activity and suppression of alpha oscillations and the 'default-mode' network. Proc. Biol. Sci. *286*, 20191016.

37. Simon, D.A., and Daw, N.D. (2011). Neural correlates of forward planning in a spatial decision task in humans. J. Neurosci. *31*, 5526–5539.
38. de Cothi, W., Nyberg, N., Griesbauer, E.-M., Ghanamé, C., Zisch, F., Lefort, J., Fletcher, L., Newton, C., Renaudineau, S., Bendor, D., et al. (2020). Predictive Maps in Rats and Humans for Spatial Navigation. bioRxiv, 2020.09.26.314815.
39. Okaichi, H. (1996). Effects of fimbria-fornix lesions on door discrimination and route choice in a lattice maze by rats. Neurobiol. Learn. Mem. *66*, 155–166.
40. Arleo, A., and Rondi-Reig, L. (2007). Multimodal sensory integration and concurrent navigation strategies for spatial cognition in real and artificial organisms. J. Integr. Neurosci. *6*, 327–366.
41. Poucet, B. (1993). Spatial cognitive maps in animals: new hypotheses on their structure and neural mechanisms. Psychol. Rev. *100*, 163–182.
42. Epstein, R.A., Patai, E.Z., Julian, J.B., and Spiers, H.J. (2017). The cognitive map in humans: spatial navigation and beyond. Nat. Neurosci. *20*, 1504–1513.
43. Mankin, E.A., Sparks, F.T., Slayyeh, B., Sutherland, R.J., Leutgeb, S., and Leutgeb, J.K. (2012). Neuronal code for extended time in the hippocampus. Proc. Natl. Acad. Sci. USA *109*, 19462–19467.
44. Grieves, R.M., Duvelle, É., Wood, E.R., and Dudchenko, P.A. (2017). Field repetition and local mapping in the hippocampus and the medial entorhinal cortex. J. Neurophysiol. *118*, 2378–2388.
45. Brotons-Mas, J.R., Schaffelhofer, S., Guger, C., O'Mara, S.M., and Sanchez-Vives, M.V. (2017). Heterogeneous spatial representation by different subpopulations of neurons in the subiculum. Neuroscience *343*, 174–189.
46. Lever, C., Burton, S., Jeewajee, A., O'Keefe, J., and Burgess, N. (2009). Boundary vector cells in the subiculum of the hippocampal formation. J. Neurosci. *29*, 9771–9777.
47. Krupic, J., Bauza, M., Burton, S., and O'Keefe, J. (2016). Framing the grid: effect of boundaries on grid cells and navigation. J. Physiol. *594*, 6489–6499.
48. Wernle, T., Waaga, T., Mørreaunet, M., Treves, A., Moser, M.-B., and Moser, E.I. (2018). Integration of grid maps in merged environments. Nat. Neurosci. *21*, 92–101.
49. Javadi, A.-H., Emo, B., Howard, L.R., Zisch, F.E., Yu, Y., Knight, R., Pinelo Silva, J., and Spiers, H.J. (2017). Hippocampal and prefrontal processing of network topology to simulate the future. Nat. Commun. *8*, 14652.
50. Spiers, H.J., and Gilbert, S.J. (2015). Solving the detour problem in navigation: a model of prefrontal and hippocampal interactions. Front. Hum. Neurosci. *9*, 125.
51. Pezzulo, G., Kemere, C., and van der Meer, M.A.A. (2017). Internally generated hippocampal sequences as a vantage point to probe future-oriented cognition. Ann. N Y Acad. Sci. *1396*, 144–165.
52. Stoianov, I., Maisto, D., and Pezzulo, G. (2020). The hippocampal formation as a hierarchical generative model supporting generative replay and continual learning. bioRxiv. https://doi.org/10.1101/2020.01.16.908889.
53. Pfeiffer, B.E., and Foster, D.J. (2013). Hippocampal place-cell sequences depict future paths to remembered goals. Nature *497*, 74–79.
54. de Cothi, W., and Barry, C. (2019). Neurobiological successor features for spatial navigation. bioRxiv. https://doi.org/10.1101/789412.
55. Grieves, R.M., Duvelle, É., and Dudchenko, P.A. (2018). A boundary vector cell model of place field repetition. Spat. Cogn. Comput. *18*, 217–256.
56. Jeffery, K.J., Anderson, M.I., Hayman, R., and Chakraborty, S. (2004). A proposed architecture for the neural representation of spatial context. Neurosci. Biobehav. Rev. *28*, 201–218.
57. Hayman, R.M., and Jeffery, K.J. (2008). How heterogeneous place cell responding arises from homogeneous grids–a contextual gating hypothesis. Hippocampus *18*, 1301–1313.
58. Singer, A.C., Karlsson, M.P., Nathe, A.R., Carr, M.F., and Frank, L.M. (2010). Experience-dependent development of coordinated hippocampal spatial activity representing the similarity of related locations. J. Neurosci. *30*, 11586–11604.
59. Derdikman, D., Whitlock, J.R., Tsao, A., Fyhn, M., Hafting, T., Moser, M.-B., and Moser, E.I. (2009). Fragmentation of grid cell maps in a multicompartment environment. Nat. Neurosci. *12*, 1325–1332.
60. Grieves, R.M. (2015). The Neural Basis of a Cognitive Map. http://hdl.handle.net/1893/21878.
61. Leutgeb, J.K., Leutgeb, S., Moser, M.-B., and Moser, E.I. (2007). Pattern separation in the dentate gyrus and CA3 of the hippocampus. Science *315*, 961–966.
62. Keinath, A.T., Nieto-Posadas, A., Robinson, J.C., and Brandon, M.P. (2020). DG-CA3 circuitry mediates hippocampal representations of latent information. Nat. Commun. *11*, 3026.
63. Sharp, P.E., Kubie, J.L., and Muller, R.U. (1990). Firing properties of hippocampal neurons in a visually symmetrical environment: contributions of multiple sensory cues and mnemonic processes. J. Neurosci. *10*, 3093–3105.
64. Garcia, D. (2010). Robust smoothing of gridded data in one and higher dimensions with missing values. Comput. Stat. Data Anal. *54*, 1167–1178.
65. Garcia, D. (2011). A fast all-in-one method for automated post-processing of PIV data. Exp. Fluids *50*, 1247–1259.
66. Kadir, S.N., Goodman, D.F.M., and Harris, K.D. (2014). High-dimensional cluster analysis with the masked EM algorithm. Neural Comput. *26*, 2379–2394.
67. Skaggs, W.E., McNaughton, B.L., Wilson, M.A., and Barnes, C.A. (1996). Theta phase precession in hippocampal neuronal populations and the compression of temporal sequences. Hippocampus *6*, 149–172.
68. Jercog, P.E., Ahmadian, Y., Woodruff, C., Deb-Sen, R., Abbott, L.F., and Kandel, E.R. (2019). Heading direction with respect to a reference point modulates place-cell activity. Nat. Commun. *10*, 2333.
69. Schmitzer-Torbert, N., Jackson, J., Henze, D., Harris, K., and Redish, A.D. (2005). Quantitative measures of cluster quality for use in extracellular recordings. Neuroscience *131*, 1–11.
70. Schmitzer-Torbert, N., and Redish, A.D. (2004). Neuronal activity in the rodent dorsal striatum in sequential navigation: separation of spatial and reward responses on the multiple T task. J. Neurophysiol. *91*, 2259–2272.
71. Holm, S. (1979). A Simple Sequentially Rejective Multiple Test Procedure. Scand. J. Stat. *6*, 65–70.

STAR★METHODS

KEY RESOURCES TABLE

REAGENT or RESOURCE	SOURCE	IDENTIFIER
Experimental models: organisms/strains		
Lister Hooded Rats	Charles Rivers Laboratories, UK	N/A
Deposited data		
Place cell and behavior data generated and used in the present study	This paper	EBRAINS: https://doi.org/10.25493/7NJQ-ANH
Software and algorithms		
KlustaKwik	http://klusta-team.github.io/klustakwik/	v.3.0
Tint	Axona, St. Albans, U.K.	V 4.4.16
MATLAB	The Mathworks	2020a
Code used in the present paper	This paper	EBRAINS: https://doi.org/10.25493/7NJQ-ANH
Other		
Axona Recording System	Axona, St. Albans, U.K.	DacqUSB
Axona drives	Axona, St. Albans, U.K.	MDR-xx
Non-Cyanide Gold Plating Solution	Neuralynx Inc., MT	N/A
NanoZ system	White Matter LLC	N/A
Super-Bond C&B	Sun Medical, Shiga	N/A
Simplex Rapid (Liquid + Powder)	Kemdent®, Swindon, U.K.	N/A

RESOURCE AVAILABILITY

Lead contact

Further information and requests for resources should be directed to and will be fulfilled by the Lead Contact, Éléonore Duvelle (e.duvelle@ucl.ac.uk).

Materials availability

This study did not generate new unique reagents.

Data and code availability

The behavioral and place cell data datasets generated in this study are available online at: EBRAINS: https://doi.org/10.25493/7NJQ-ANH. The MATLAB code generated and used for this study is available online at: EBRAINS: https://doi.org/10.25493/7NJQ-ANH.

EXPERIMENTAL MODEL AND SUBJECT DETAILS

Five Lister-Hooded male rats weighing approximately 300-600 g and aged 3-6 months at the start of the experiment were used. They were first housed in pairs at 20 ± 2°C under a 12/12h light/dark cycle starting at 12 AM. They were provided with *ad libitum* water, food, environmental enrichment and daily handling from the experimenter. After implant surgery, rats were housed individually and allowed to recover for one week before food-restriction started to maintain their weight at 90%–95% of free-feeding body weight. One of the rats had prior experience with a spatial task in the same experimental room and another one had prior experience with a linear track task in a different room; the other 3 rats were naive. All procedures complied with the national [Animals (Scientific Procedures) Act, 1986 United Kingdom] and international [European Communities Council Directive of 24 November 1986 (86/609/EEC)] legislation governing the maintenance of laboratory animals and their use in scientific experiments.

METHOD DETAILS

Overall summary

Upon arrival in the rat colony, rats were handled daily for at least a week, implanted and allowed to recover for another week. They were then food-restricted, trained, and screened for place cells. Once rats had passed all training phases and signals from putative place cells (> 4 simultaneous cells) were detected, they were recorded in two different sequences (1 per day), in baseline conditions (all doors open) and test conditions (1 door locked both ways or all doors locked only one-way). After successful recordings in each

sequence type, the tetrodes were lowered in an attempt to detect new cells and recordings were repeated in each phase, usually with a new door condition (e.g., a different door was locked or all doors were locked the other way); this process continued until fewer than 4 cells were detected simultaneously. Rats were then perfused transcardially with saline followed by 9% formalin and their brains were extracted and stained to confirm the location of recording tetrodes.

Room and experimental environment

The experimental room was equipped with distinct distal visual cues on each wall and dim ceiling lighting. A schematic of the apparatus and room cues as well as photos is shown in Figure S1A. The experimenter, the recording system and the computer desk were in the same room. The custom-built recording apparatus consisted of four 60x60 cm gray-painted wooden boxes connected to each other via four 16x16 cm dark gray door systems. Each box also had two 'dummy doors' (same dimensions and color as the real doors, but made of one panel instead of two) appended to their external sides (Figure 1A). All walls were 20 cm high and were made of painted hardboard with small (~4mm diameter) perforations. On the corner of each box, a bell was placed which could be activated remotely by pulling a string from the experimenter's desk. The bell system acted as a sound cue to inform the rat of the next rewarded box. The door mechanism consisted of two vertical panels glued to a rotating wooden rod and equipped with one spring each, to ensure that the door would remain shut unless it was being pushed. Curved black plastic stripes were added on the top of the doors to guide the recording cables. A slot present at the top of each door panel allowed for the insertion of 4 small metallic locks that could block the panel on one or both sides. Since the locks were inserted at the top, the rats could not see them unless they were in a rearing position. Thus, the doors would look and feel the same from a rat's point of view whether locked or not, the only difference being that locked doors could not be pushed open. Note that we indifferently use "locked" or "closed" to mean a door that cannot be pushed. The 4-room environment was placed on top of 60 cm-high cardboard boxes. A padded headcap was added around the drives and headstages to help rats with door-pushing and absorb possible shocks on the implants. An overhead camera centered on the environment provided video input to the tracking system. Tracking and electrophysiology data were collected using a 64-channel recording system (DacqUSB, Axona, St. Albans, UK). During screening, recording and most of the training, the animal was connected to the recording system via 4 flexible cables attached to the ceiling by elastic bands.

An elevated rotating platform (80 cm high) was placed next to the environment where rats could rest before and after screening / training / recording sessions. Screening sessions (i.e., monitoring brain signals to decide whether tetrodes were in the hippocampal cell layer or not and move the tetrodes accordingly) were run in a plastic 120x120 cm black square with 20 cm high walls placed on top of the 4-room environment.

Task

The final task, used during all recordings and most of the training, consisted of separate **sessions** which each contained an exploration phase followed by several 'trials' starting at a bell sound and ending before the next bell sound. The food reward used during the experiment was either rice krispies / coco pops (Kelloggs, Michigan, US) or rice pops / choco pops (Waitrose and partners, London, UK). The reward type could change across days but would remain the same throughout a given recording sequence.

During the **exploration phase**, the rat was placed in a given compartment with no food provided and allowed to explore all 4 boxes. After this, the **task trials** started. On a given trial, the bell of a specific box was rung and food was thrown there by the experimenter; once the rat reached the rewarded box, more food would be thrown or placed in the box at regular intervals. During this specific step, the experimenter would sometimes come closer to the box to place food in specific places; attention was drawn to placing food in front of doors (real or dummy), in corners, and in any under-sampled locations. The 'trial' ended after a given time passed in the same box (at least 30 s, excluding time spent out of the rewarded box). Then, a new trial started using the next box, selected from a predetermined goal list, until all boxes of the sequence had been visited. The **goal list** was created pseudo-randomly by the experimenter with the following constraints: each box (A, B, C and D, Figure 1B) should be used 3 times as goal, making up 12 trials in total; all four boxes should be used before repeating a box; distances between successive boxes should vary. An example goal list would be ABDC BACD CBAD. A new list was generated for each recording sequence.

A daily recording **sequence** was divided into 5 sessions, each using the same goal list. The first two sessions were considered the **baseline**, with all four doors open both ways. The next two sessions were the **test sessions**, using either a 'closed-door' configuration (1 door locked both ways) or a 'one-way' configuration (all 4 doors open only one way). The last session of the sequence was back to baseline, i.e., all doors open. In between sessions, the rat was placed on the elevated platform with access to water and allowed to rest for 5 or 10 min. During this time, the experimenter manipulated all 4 doors to minimize possible olfactory biases and locked or opened the appropriate doors.

In summary, there were two possible sequences:

'Closed-Door' sequence = all open, all open, closed, closed, all open (Figure 1B).
'One-way' sequence = all open, all open, one-way, one-way, all open (Figure 1C).

A 'Closed-Door' sequence was generally followed on the next day by a 'One-Way' sequence or was repeated with a different locked door. Tetrodes were advanced by at least ~25μm to sample new cells only when a rat had successfully completed one of each sequence type. In some cases the same sequence type was recorded without advancing the tetrodes, meaning that the same sample of place cells could have been recorded, but the order of rewarded boxes and the chosen locked door(s) were always

different in each session. The maze was cleaned with alcohol spray between animals, and urine traces or faeces were locally cleaned on the rare occasions when they appeared during the experiment or when detected between sessions. No attempt was made to remove other possible olfactory cues left by the rats between sessions of a recording sequence.

Training

All rats were pre-trained post-surgery in the four-room environment for 2 to 5 sessions every weekday, as detailed below. Two of the rats were also used before implant surgery to pilot the door system, one of those in a different room and apparatus. Training in the 4-room took 6 to 15 days (median = 10 days) depending on how fast the rats reached the final criteria; during this they were also screened for hippocampal cells. Training aimed to familiarise rats with: i) pushing doors, ii) foraging efficiently in each box and iii) running for food for long periods of time (the experiment lasting on average 2.5h each day). No explicit training of the association between the goal and the bell sound was done. Undesirable behaviors like climbing on walls or doors and chewing maze parts were discouraged from the start of training by either making a loud clapping noise or pushing the rats. Training data were not analyzed. Training followed the three following phases:

Familiarisation

Rats were allowed to freely explore the maze without food. After 5 min, rats were taken out of the environment and placed onto the elevated platform. This was repeated once per box.

Door training

With food present in all 4 boxes, rats were released in a random box and taken out of the environment after 10 min or once all food had been eaten, whichever came first. After 5 min, if the rat hadn't gone spontaneously through a door, the experimenter would encourage door-pushing (e.g., going through a door with a hand, holding the door half-open, attracting the rat to the other side of a door with noises or food). This phase was repeated until rats were able to easily use doors to move from box to box. Interestingly, most rats started to use the doors spontaneously from early maze exposure.

Task learning

Rats were plugged in to the recording system from the third session of this phase. Here, rats were doing the task as described above (STAR methods - Task), except that each training session lasted 20 min maximum. For three of the rats (r35, r37 and r38), locked doors (either one closed door or one one-way door) were introduced once they had mastered the baseline condition of the task. For the other two rats, closed-door sessions were added every 2-4 'all-open' sessions. For all rats except r35, only two of the doors were used as closed or one-way doors during training so that the other two, new doors could be closed in priority during the actual recording. Rats were considered ready for recordings once they could do 11 trials (as defined in STAR methods - Task) in 20 min for two out of the last three training sessions, except for r44 for whom the criterion was 11 trials in 25 min. Rats' behavior was generally quite disturbed upon their first closed-door encounter (i.e., rats might try to pull on doors, push harder, climb, or avoid all doors altogether) but stabilized after a few more encounters. Sequences where rats stopped performing the task before completing the full sequence were excluded from the analysis (8 Closed-Door and 1 One-Way excluded).

Microdrive and implantation surgery

Axona drives (MDR-xx, Axona, UK) were loaded with 8 tetrodes made of four 17 μm of diameter platinum-iridium wires (California Fine Wire, Grover Beach, CA). Tetrode tips were gold-plated (Non-Cyanide Gold Plating Solution, Neuralynx Inc., MT) using a NanoZ system (White Matter LLC) to reduce the impedance to 180–250 kΩ at 1kHz. Two drives were implanted on each rat (one per hemisphere), above the CA1 field of dorsal hippocampus, using standard stereotaxic procedures under isoflurane anesthesia and sterile conditions. The coordinates relative to Bregma were as follows: AP: −3.5 to 4.0 mm; ML: ± 2.4mm; DV: 1.3 to 1.5 mm from dura surface. Both drives shared the same ground wire, connected to a ground screw above the cerebellum. 6 jewellers' screws helped anchor the drive to the skull, together with one layer of Super-Bond C&B (Sun Medical, Shiga) followed by several layers of dental cement (Simplex Rapid, Kemdent®). A long-acting analgesic (Carprofen) and saline solution were given subcutaneously at the start of surgery. Post-surgery, rats were provided with another analgesic (Meloxicam) in their food for 3 days.

Screening and recording

Daily or twice-daily screening sessions (spaced by at least 4h) started 1 week post-surgery, during which the animal rested on the elevated platform then foraged for the same reward used in the experiment in the square plastic environment for 8-16 min. Signals were screened for signs of sharp-wave ripples and pyramidal cells. If no hippocampal activity was detected, tetrodes were lowered by approximately 25 or 50 μm. Extracellular activity was collected with DacqUSB, the signal was first sampled at 50kHZ, amplified then band-pass filtered between 300 and 7000 Hz, digitized at 48 kHz and could be further amplified 10–40 times at the experimenter's discretion. Local Field Potential (LFP) data was obtained by sampling signals from selected channels at 4.8 kHz. Note that LFP data was not used in the current study.

Histology

When no more than 4 putative CA1 signals were observed in a given rat and sharp-wave ripple amplitude was observed to decrease on both drives, small electrolytic lesions were created by passing a positive current (5 μm for 10 s) through chosen electrodes while the animal was deeply anaesthetised. The rats were then overdosed with pentobarbitone before being transcardially perfused with a saline solution followed by a formalin solution (10%). The brains were preserved in formalin for at least 2 days, then, optionally into a 30% sucrose solution for another 2-3 days. Brains were then frozen and sectioned in 40 microns slices stained with Cresyl Violet. The electrode tracks and lesions signs were detected under a microscope to confirm recording sites. All recording sites used in the analysis were confirmed to be in hippocampus CA1 on both hemispheres (see Figures S1B and S1C.

Position tracking

The head position of animals was tracked continuously at 50Hz, using an infrared LED affixed to the recording headstage and custom tracking software (DacqTrack, Axona Ltd., St. Albans, UK). For segments of missing tracking data, we simultaneously interpolated and smoothed the existing data using an unsupervised, robust, discretized, n-dimensional spline smoothing algorithm (MATLAB function *smoothn*[64,65]). For each sequence of sessions, we manually fitted a wire-frame to the position data, from which we extracted the maze boundary and doorway positions.

Event flags

Experimenters recorded behavioral events such as door pushes (whether they led to a door-crossing or not) and bell sounds online during recording by pressing on a miniature wireless keyboard. The recording system stored the time and type of keypress synchronized with neural and position data. Although the majority of these manual event flags were correct and utilized in priority, incorrect flags were corrected programmatically offline based on the animal's tracked position in combination with trial-specific data. These corrections apply only to the behavioral measures related to bell sounds (STAR methods - Response to bell sounds), correct door pushes and correct foraging (STAR methods - Correct door pushes and correct foraging). Bell sound events recorded by the experimenters were never corrected. However, a minority of trials were rejected because the rat was tracked in the rewarded compartment synchronously with the bell sound and thus no first door push or foraging choice could be determined. In further trials a door push was recorded that was not possible given the animal's location and these were replaced with the first door through which the animal moved after the bell sound. The majority of these two error types were due to short time lags between the animal's behavior and registering the event flag key press. Lastly, in a subset of trials, no door push was recorded and the missing value was filled using the first door through which the animal moved after the bell sound.

Behavior discrimination

Animals engaged in two different modes of behavior which we termed 'foraging' or 'goal-directed'. When animals moved through the maze in a fast and direct way (for instance after a bell sound but before they reached the food), marked by rapid and direct locomotion, we classified the behavior as goal-directed. Other periods were spent freely searching for food (for instance after arriving in a rewarded box) and were marked by slower and circuitous locomotion, this was categorised as foraging. These two behavioral modes were automatically categorised. For each visit an animal made to a box, it was categorised as foraging if *i*) the distance covered in the visit was more than 120cm and *ii*) the animal covered more than 20% of the box area during the visit.

Distance was calculated as the total distance traveled along the visit path in non-overlapping 1 s windows, coverage area was calculated by dividing boxes into 100 bins (unsmoothed) and counting the proportion of bins containing more than 1 position data sample. For comparison, the minimum distance between two doorways along a circular arc would be approximately 50cm and cover approximately 10% of a box. Conversely, rats were considered to be in a goal-directed mode if: *i*) moving through a door (1 s before to 1 s after), *ii*) pushing on a door (2 s before to 1 s after) or *iii*) not foraging.

Response to bell sounds

To test whether rats were aware of and responded to the bell sounds, we first compared their running speed in the period 1 s before and 1 s after each bell sound. Instantaneous speed was calculated for every position data point as the total distance traveled divided by time passed.

Next, we looked at the time between a bell sound and the next door push and compared these values to a shuffle. For the shuffle we generated N random time points uniformly throughout each session where N was the number of real door push events. We then calculated for each bell sound the time between that and the next random time point. This procedure provides the time expected between bell sounds and door pushes if the two events were completely dissociated.

Correct door pushes and correct foraging

We next sought to determine whether rats navigated to rewarded boxes using optimal paths. For each session we generated a graph with directed edges (MATLAB *digraph*) representing the nodes and possible routes given the maze structure and connectivity. Using this graph, we calculated the minimum number of doors that would need to be crossed when moving between any two boxes and the optimal door sequence that would need to be used. For example, with all doors open the minimum distance between boxes A and B would be 1: moving through the door directly between them. However, if this door was locked the minimum distance would instead be 3 as the rat would have to travel via boxes D and C.

With this approach, for every bell sound we looked at the first door push the rats made following the bell and assessed whether this door belonged to the optimal path to the rewarded box given the maze connectivity. We then analyzed the performance of rats depending on the distance from the start box to the goal box. In this analysis, when all doors were open (sessions O1, O2 and O3) we discarded trials where the rat was diagonally opposite the correct box (distance of 2) as in this case both doors and paths would be equally optimal. Similarly, in the open sessions the rat could never be 3 or more doors away from the correct box. In the sessions with a connectivity change (C1 and C2), optimality at all possible distances was assessed.

Regardless of the route taken after a bell sound we also sought to determine whether rats preferentially started to forage in the rewarded box. For this we looked for the first box in which the rat foraged after each bell sound (STAR methods: Behavior discrimination) and assessed whether this was the rewarded box or not. This analysis was independent of the optimality analysis described above, meaning that a rat could take a non-optimal path or push on locked doors but still forage first in the rewarded box.

Neural activity analyses

Single-unit data were first processed using an automated spike-sorting algorithm (Klustakwik v3.0,[66]) using the first three principal components and peak waveform amplitude as parameters. Manual refinement of the classification was then done using the TINT spike-sorting software (Axona, St Albans, UK). Only well-isolated putative pyramidal cells were kept (pyramidal-like waveforms, no or few spikes in the refractory period).

Firing rate maps and spatial information

Spike and dwell time maps were constructed as bivariate histograms of the spike and position data respectively (2cm square bins, MATLAB: *hist3*) after speed-filtering the data to remove periods where the animal's running speed was less than 5 cm/s (to avoid contamination of the data by possible reactivation events). These maps were then smoothed with a two-dimensional Gaussian kernel (standard deviation: 2.5 bins, kernel size: 9 × 9 bins, MATLAB: *imgaussfilt*). Firing rate maps were calculated by dividing spike maps by the corresponding dwell map for that session. In all maps, bins visited by the animal for less than 0.05 s were considered empty. This procedure was repeated using *i*) all spike and position data, *ii*) data filtered to include only foraging behavior and *iii*) data filtered to include only goal-directed behavior, always using only speed-filtered data (STAR methods: Behavior discrimination).

Spatial information content in bits/second was calculated using a method reported previously[67] as:

$$\textit{spatial information} = \sum_{i=1}^{N} p_i \frac{\lambda_i}{\lambda} \log_2 \frac{\lambda_i}{\lambda}$$

where the environment is divided into non-overlapping spatial bins $i = 1,\ldots, N$, p_i is the occupancy probability of bin i, λ_i is the mean firing rate for bin i, and λ is the overall mean firing rate of the cell. We compared these spatial information values to a shuffle distribution using a method reported previously[68]; briefly, spike sequences were time-shifted by a random time interval (between 20 s and the duration of the session minus 20 s) in a circular manner. This method maintains their temporal order and total number while decoupling the spike times from the animal trajectory. For each shuffle we reconstructed a firing rate map and recalculated its spatial information content as described above. This procedure was repeated 100 times for each neuron. A neuron was defined as significantly spatially modulated if the observed spatial information content exceeded the 95th percentile (MATLAB: *prctile*) of its shuffled distribution.

Whenever activity between compartments was compared (e.g., place field repetition analysis, doorway rate map correlations analyses), the rate maps for individual boxes were smoothed separately to avoid spuriously smoothing data between neighboring boxes. We constructed box-specific maps by cutting position and spike data to within the boundary (minimum enclosing rectangle) of each box. Firing rate maps were then constructed as described above. From these box-specific maps we also extracted 25cm radius regions around each doorway which were used in the doorway specific analyses. In all other cases, i.e., when whole maps of the environment were compared to each other, the map was smoothed as a whole.

Unit classification

Units were classified as putative place cells if their mean firing rate was greater than 0.1Hz in at least 2 sessions and if, in the session with the highest firing rate:

1. Average firing rate was greater than 0.1 Hz and less than 5 Hz and;
2. Spatial information content was greater than 0.5 bits/second and;
3. Spatial information content exceeded the 95th percentile of a spike train shuffle and;
4. The cell's width of waveform (peak to trough) was greater than 300μs.

A cluster's width of waveform was defined as the time between its maximum and minimum amplitude (peak to trough). We extracted this value from the channel with the greatest amplitude in the session with the highest firing rate. All further analyses were conducted only on those cells passing these criteria, which aimed at removing possible interneurons, silent cells and non-spatial cells. Note that obvious interneurons (high-firing clusters with small width of waveform) were removed at the spike-sorting stage as we were focusing on place cells, and interneurons are generally too heterogeneous to analyze. Cells were analyzed separately in the two sequences of conditions (which may contain repeated recordings of the same cell). Within the same condition, it is possible

CellPress
OPEN ACCESS

that the same cell was recorded more than once, although we would often lower the tetrodes by at least 25μm between two recordings of the same type and we manipulated a different door in consecutive recordings of the same type. Finally, all analyses were repeated on one session per rat – the one with most simultaneously recorded cells - with similar results and conclusions (data not shown).

Rate map correlations

We correlated the spatial activity of place cells at three different levels of specificity, between sessions, between boxes and between doorways. All correlations were pairwise Pearson correlations (MATLAB *corr*) and we only correlated a pair of firing rate maps when at least one map had a peak firing rate greater than 1Hz[4]. Using this process we compared every session to every other session within a sequence, every box to every other box within a sequence and every doorway to every other doorway within a sequence.

For session comparisons we extracted the correlations between sessions O1 and O2 (first two sessions with all doors open) which gives a baseline measure of the stability of place cells in an unchanging environment. We then extracted the comparisons between O2 and C1 (the last open door session and the first closed door session); if cells changed their firing in response to a change in connectivity we would expect these correlations to be lower than the baseline. We also looked at the change in firing over time by extracting correlations between sessions separated by increasing durations (i.e., O1 and O2 are consecutive while O1 and O3 are separated by 3 sessions or approximately 90 minutes).

We also looked more specifically at activity around the doorways. For this we looked at each side of every door separately and we categorised these into two groups: the 'closed/control' group consisted of the doors that were locked during sessions C1 and C2, the 'open' group consisted of all other doors. Note that in the One-Way sequence a single doorway will contribute one side to the closed/control group and one side to the open group. Next, we extracted the correlations between sessions O1 and O2 separated into closed/control and open doorway values. These were then averaged so that every cell contributed one value to each group. As before, this acts as a baseline and we compared it to the same values extracted from correlations between sessions O2 and C1.

Individual remapping between sessions

To more broadly categorise remapping between the different sessions, for every individual cell we looked at if, where and when remapping occurred between consecutive sessions. First, for each place cell we correlated the firing rate maps for consecutive sessions (O1 & O2, O2 & C1, C1 & C2 and C2 & O3). Remapping was defined as a change of spatial firing pattern at an above-chance level where chance was determined using a shuffle procedure. For this, for every sequence of sessions with more than 10 simultaneously recorded place cells (12 sequences for Closed-Door, 9 for One-Way) we correlated the rate maps of random cells between each session and the next, a thousand times. These shuffled distributions were very similar and thus combined to give four distributions, each one describing the chance of remapping between consecutive sessions. We then compared the between-session correlation values of all place cells to these combined shuffles; when the value for a given cell was lower than the 95th percentile of the corresponding shuffle we considered that it had remapped between those two sessions. The rationale for shuffling within sequences was to ensure cells were only compared to others recorded under the same behavioral constraints. For every cell, this procedure yields four binary outcomes (remapping or no remapping between O1 & O2, O2 & C1, C1 & C2 and C2 & O3). For visualization we generated a histogram of all 16 possible combinations.

Foraging versus goal-directed remapping

After differentiating foraging from goal-directed behavior (STAR methods - Behavior discrimination) we sought to compare the firing of place cells between these two states. First, for each place cell we correlated its foraging and goal-directed firing rate map in each session (O1, O2, C1, C2 and O3). Remapping was defined as a change of spatial firing pattern between these at an above-chance level where chance was determined using a shuffle procedure similar to that described above (STAR methods - Remapping between sessions), but comparing foraging maps to goal-directed maps of random cells within the same session. We then compared the distribution of observed correlation values from all place cells to these combined shuffles for each session. If the median observed correlation value fell above the 95th percentile of the corresponding shuffle distribution we considered that cells were more stable between foraging and goal-directed modes than chance.

Firing rate remapping

In addition to the correlation-based analyses described in the main text, we also looked at rate remapping (changes in place cell firing rate independent of spatial changes) using a method described previously[24]. Briefly, this followed the same procedure as the correlation analyses but using the following difference metric in place of the Pearson r correlation:

$$Fuhs = \frac{\sum_x |f_1(x) - f_2(x)|}{\sum_x |f_1(x)| + |f_2(x)|}$$

where the variable x ranges over map locations, and $f_1(x)$ and $f_2(x)$ are two place field firing rate maps that have been zero-normalized by subtracting off their respective mean firing rates. As outlined by Fuhs and colleagues[24], the metric calculates the ratio of the difference between rate maps to the difference between each rate map and its mean. Thus, the metric equals 0 for identical fields and approaches a maximum of 1 when maps differ either spatially or in terms of firing rate. We used this 'Fuhs metric' to compare firing

rate maps between sessions and only for those place cells which were found not to significantly spatially remap between any sessions (see STAR methods - Remapping between sessions).

Place fields

When detecting place fields, we used smoothed, speed-filtered (> 5cm/s) firing rate maps generated only from foraging data, as described in STAR methods: Firing rate maps and spatial information. Firing rate maps were thresholded to 20% of their maximum value, place fields were defined as regions within the thresholded maps with *i)* an area greater than 9 contiguous bins and *ii)* a peak firing rate greater than 1Hz.

For each field we extracted its area (number of bins in the region), weighted centroid (center of the region based on bin locations and firing rates), convex hull (coordinates of minimum enclosing polygon) and mean firing rate (average bin value, all values calculated using MATLAB *regionprops*).

Place field repetition

To quantify the prevalence of place field repetition, for every place cell we calculated the average correlation between all possible pairs of boxes for each session independently. We compared the resulting distributions to chance, where chance was determined using a shuffle procedure similar to that described above (STAR methods - Remapping between sessions) but comparing box firing rate maps to box maps of random cells within the same session. Additionally, for session O2 only, we repeated this procedure after dividing place cells into groups based on the number of place cells they exhibited in this session.

For all sessions the distribution of observed correlations differed from the shuffles and was significantly shifted toward 1. This result suggests greater place field repetition than chance but does not quantify the degree of place field repetition. For this we designed shuffles to test if the observed values reflected place field repetition in 2, 3 or 4 boxes. For each shuffle we took each cell in turn and collected all 4 compartments for session O1. We next selected a random compartment and duplicated this across multiple compartments (2, 3 or 4 depending on the shuffle type). For the duplication we took the rate maps for that compartment across the different sessions. For example, a shuffle designed to reflect repetition in two compartments might include compartment A, B and C in session O1 and compartment A from session O2. Our reasoning was that the same compartment sampled in different sessions provides a good approximation for sampling the same field in multiple compartments (assuming place field repetition).

Place field overrepresentation

Next, to test if more or fewer fields were found around the locked doors, for each session we found the total number of fields with their centroid less than 25cm from each door. For the Closed-Door sequence we then calculated the average number of fields around the 3 open doors and compared this to the number of fields around the locked door. If these were equally represented we would expect 50% of this total to fall around each door type in each session, which we tested using a chi-square test of equal proportions (custom MATLAB function, *p-value*s were corrected across sessions using the Holm-Bonferroni method). For One-Way sequences we used a similar procedure except that we compared the number of fields within 25cm of the locked sides of the doors to the open sides.

To test more generally if doorways were overrepresented relative to the boxes and dummy doors, for each session and for each sequence type separately we counted the number of fields falling within 25cm of any door, any box center or any dummy door and expressed these values as proportions of the total of the three (N). We then calculated the total surface of each test area by taking the median across all dwell maps and thresholding the resulting map to discard bins with a median of less than 0.01 s. Surface area was then calculated as the total remaining bins within 25cm of the test areas. The expected proportion of fields for each test area was then estimated as the proportion of total surface area in the dwell map included in this test area multiplied by N. Lastly, we tested the observed proportions against the expected proportions using a chi-square test of equal proportions (custom MATLAB function as above).

Place field firing rate and area

To further test if field properties such as mean in-field firing rate and total field area (STAR methods: Place fields*)* changed in response to a change in connectivity we extracted all place fields with a weighted centroid within 25cm of a doorway and calculated average values for each session.

Next, we separated fields into 2 groups: those with a weighted centroid within 25cm of a door (or side of a door in the One-Way sequence) which remained open throughout the sequence and those within 25cm of one which was locked during the sequence. For visualization we rotated fields in the Closed-Door sequences so that the locked doorway was always at the bottom and for the One-Way sequences we flipped the fields along the x axis so that fields on the locked or open side of a door would overlap. For each spatial bin we then counted the total number of overlapping fields observed in that bin and plotted the result as a density heatmap. Finally, we calculated the average area and mean in-field firing rate for each group in each session and sequence.

Place field extent across doors

To test if fields that extended across doorways continued to do so when these doors were locked we found all of the fields within 25cm of a door that was locked in C1. We then found the area (total bins) found on each side of the door (a and b) and calculated a ‘bridge index’ as:

$$bridge\ index = 1 - \left|\frac{a-b}{a+b}\right|$$

This index varies from 0 when a field's area is entirely on one side of the door to 1 when a field's area is equally distributed on each side of the door. For visualization we then projected the field convex hull onto an axis perpendicular to the locked door and extracted the maximum limits of this projection and the centroid along this axis.

Bayesian decoding of box quadrants

Position decoding was performed using a memoryless Bayesian algorithm that assumes spiking is Poissonian, independent across neurons, and compares the spiking vector of simultaneously recorded cells to their expected firing rates given by their rate maps[27,28]. We used rate maps constructed from O1 when decoding position from neural activity in session O2 and O2 rate maps were used for C1 decoding, meaning the 'test' and 'training' data always belonged to different datasets.

The position and spike data of place cells were discretized into τ = 300 ms windows. Windows in which the animal had a velocity less than 5 cm/s were removed to reduce contamination by the non-local reactivations that can arise during hippocampal replay. Only sessions with at least 15 simultaneously recorded place cells were considered (Closed-Door, n = 9; One-Way, n = 3). The number of spikes that occurred in a given cell during a decoding window is denoted as σ_i and thus the vector of spiking activity for all simultaneously recorded cells is $\overline{\sigma} = \{\sigma_1, \sigma_2, \sigma_3, \ldots\}$. For every position x and place cell i, the expected value for the firing rate λ_x^i is retrieved from the rate map. Using the Poissonian assumption, the probability of observing spike vector $\overline{\sigma}$ given x is:

$$P(\overline{\sigma}|x) = \prod_i P(\sigma_i|x) = \prod_i Poiss(\sigma_i|\lambda_x^i\ \tau)\ where\ Poiss(\sigma|\lambda) = \frac{e^{-\lambda}\lambda^{\sigma}}{\sigma!}$$

And via Bayes' rule:

$$P\left(x|\overline{\sigma}\right) = \frac{P\left(\overline{\sigma}|x\right)P(x)}{P(\sigma)}$$

By using a uniform prior distribution $P(x)$, and enforcing $\sum_x P(x|\overline{\sigma}) = 1$, the decoded position becomes:

$$Decoder(\overline{\sigma}) = argmax_x\ P(x|\overline{\sigma}) = argmax_x \prod_i Poiss(\sigma_i|\lambda_x^i\ \tau)$$

The decoded positions and true positions were assigned to a box and a quadrant using a set of inequalities based on linear equations centered around the centroid coordinates of the compartments. For each session we generated a 16 × 16 confusion matrix of actual versus predicted locations, wherein each cell is populated using:

$$Confusion[i,j] = \sum_{k=0} (i == \ true\ quadrant_k)\ and\ (j == \ decoded\ quadrant_k)$$

A mean confusion matrix was computed for each session type (O2 Closed-Door, O2 One-Way, C1 Closed-Door, C1 One-Way). The rows of the session confusion matrices were converted to probability distributions by dividing by the sum of the row.

Field distance to doorway

To track the position of place fields across sessions (Figure S5) we applied k-means clustering to the field weighted centroids (MATLAB *evalclusters* with gap evaluation at [1...N] clusters where N was the greatest number of unique fields observed in a session). When analyzing the shift of fields between sessions we excluded fields that moved more than 60cm (the side length of a box). We tested if fields shifted their positions relative to the control or locked door between sessions. To test this, we calculated the distance between every field and every doorway. For Closed-Door sequences, we calculated the mean and standard deviation of the distance to the closest 16 fields around the three unchanging doors and compared this to the mean and standard deviation of the distances to the closest 16 fields around the locked door. For One-Way sequences, we calculated the mean and standard deviation of the distance to the closest 8 fields on the closed side of each door and compared this to the mean and standard deviation of the distances to the closest 8 fields on the open side of each door.

Cluster quality measures

Cluster quality (Table S2) was estimated by calculating isolation distance[69,70], L_{ratio} and peak waveform amplitude, taken as the highest amplitude reached by the four mean cluster waveforms in the session with the highest firing rate. For cluster C, containing n_C spikes, isolation distance is defined as the squared Mahalanobis distance of the n_C^{th} closest non-C spike to the center of C. The squared Mahalanobis distance was calculated as:

$$D_{i,C}^2 = (x_i - \mu_C)^T - \sum_C^1 (x_i - \mu C)$$

where x_i is the vector containing features for spike i, and μ_C is the mean feature vector for cluster C. A higher value indicates better isolation from non-cluster spikes. The L quantity was defined as:

$$L(c) = \sum_{i \notin C} 1 - CDF_{\chi^2_{df}}\left(D^2_{i,C}\right)$$

where $i \notin C$ is the set of spikes which are not members of the cluster and $CDF_{\chi^2_{df}}$ is the cumulative distribution function of the distribution with 8 degrees of freedom. The cluster quality measure, L_{ratio} was thus defined as L divided by the total number of spikes in the cluster.

QUANTIFICATION AND STATISTICAL ANALYSIS

All statistics were performed in MATLAB (The Mathworks, 2020a). Omnibus tests included two-way repeated-measures ANOVAs (MATLAB *ANOVAN* with repeated-measure included as a random variable) and two-way ANOVAs (MATLAB *ANOVAN*). The most common two-way repeated-measures ANOVA design consisted of door type (closed/open) as the between subject variable, session (O1, O2, O3 …) as the within subject variable and animal as the repeated-measure. Example responses included door pushes or correlations. These tests included two-way interactions. Two-way ANOVA designs were similar but without the repeated-measure; often these were used for electrophysiology data containing missing values (i.e., cells not active in every session). In all cases, post hoc tests were based on estimated marginal means and corrected using the Tukey-Kramer method (MATLAB *multcompare*). For post hoc tests the repeated-measure was treated as a fixed instead of random variable but we only considered comparisons between groups and not across the repeated-measure of sessions.

Other tests included one-way ANOVAs (MATLAB *anova1*), one- and two-sample t tests (MATLAB *ttest* and *ttest2*), two-sample Kolmogorov-Smirnov tests (MATLAB *kstest2*) and chi-square tests of expected proportions. Chi-square tests were calculated as:

$$X^2 = \sum_i \frac{(O_i - E_i)^2}{E_i}$$

where O are the observed values and E are the expected values. The degrees of freedom, c, are N-1 where N is the number of expected values; p was calculated as one minus the value of the Chi-square distribution at X^2 with degrees of freedom c (MATLAB *chi2cdf*).

All tests were two-sided unless otherwise stated. Where multiple t tests or chi-square tests were used to compare grouped values to chance we controlled the family-wise error rate by correcting the p values using the Holm-Bonferroni method[71]. Briefly, p values were ranked in ascending order and each value was then compared to a corresponding cutoff calculated as:

$$cutoff = \frac{\alpha}{n - rank + 1}$$

where α was the target significance threshold which was always set to 0.05 and n was the total number of p values to correct. Each p value was then compared to this cutoff in turn, the first p value that exceeded its cutoff and all following p values were considered non-significant and corrected to 0.99.

Current Biology, Volume *31*

Supplemental Information

Hippocampal place cells encode global location but not connectivity in a complex space

Éléonore Duvelle, Roddy M. Grieves, Anyi Liu, Selim Jedidi-Ayoub, Joanna Holeniewska, Adam Harris, Nils Nyberg, Francesco Donnarumma, Julie M. Lefort, Kate J. Jeffery, Christopher Summerfield, Giovanni Pezzulo, and Hugo J. Spiers

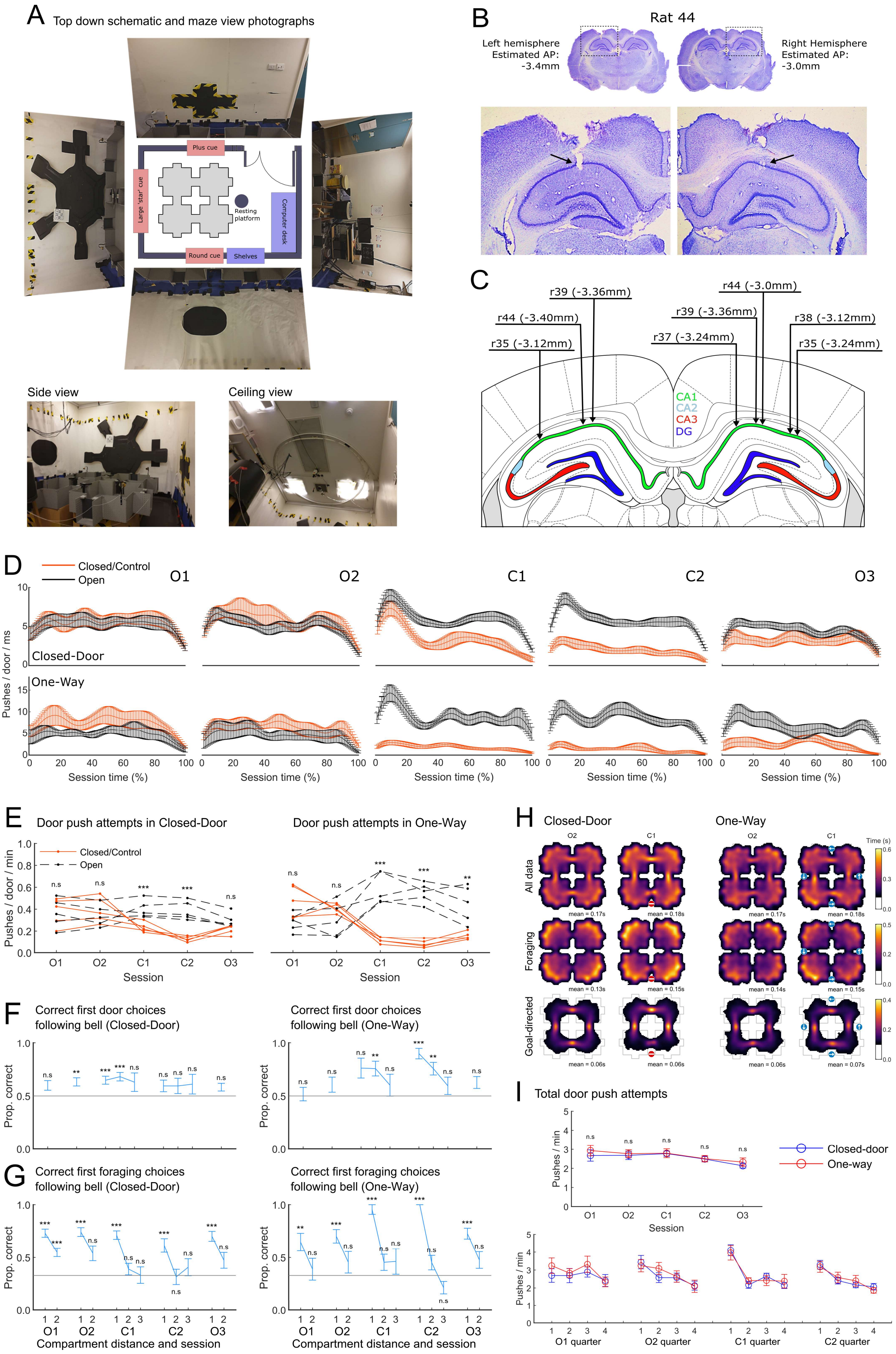

A Top down schematic and maze view photographs
Plus cue
Large 'star' cue
Resting platform
Computer desk
Round cue
Shelves
Side view
Ceiling view
B
Rat 44
Left hemisphere
Estimated AP:
-3.4mm
Right Hemisphere
Estimated AP:
-3.0mm
C
r39 (-3.36mm)
r44 (-3.0mm)
r44 (-3.40mm)
r39 (-3.36mm)
r38 (-3.12mm)
r35 (-3.12mm)
r37 (-3.24mm)
r35 (-3.24mm)
CA1
CA2
CA3
DG
D
Closed/Control
Open
O1
O2
C1
C2
O3
Closed-Door
One-Way
Pushes / door / ms
Session time (%)
E
Door push attempts in Closed-Door
Door push attempts in One-Way
Closed/Control
Open
Pushes / door / min
Session
F
Correct first door choices
following bell (Closed-Door)
Correct first door choices
following bell (One-Way)
Prop. correct
G
Correct first foraging choices
following bell (Closed-Door)
Correct first foraging choices
following bell (One-Way)
Prop. correct
Compartment distance and session
H
Closed-Door
One-Way
All data
Foraging
Goal-directed
Time (s)
I
Total door push attempts
Pushes / min
Session
Closed-door
One-way
O1 quarter
O2 quarter
C1 quarter
C2 quarter

Figure S1: Experimental Room, histology and supplementary behavioural results, related to Figure 1. Here and elsewhere, error bars indicate standard error of the mean and *:p < .05, **: p < .01, ***: p < .001. **(A)** (Top) Top down schematic of the experimental room. The maze is shown in grey. Surrounding this schematic are photos taken from the perspective of the maze along each major axis. (Bottom) Photographs of the maze and ceiling. **(B)** (Top) histological slices for one example rat showing the position of the left and right hemisphere electrodes. Text gives the estimated position of the electrodes relative to Bregma in the anterior-posterior (AP) axis. (Bottom) Close-up view of the dotted region. Arrows denote the position where the electrodes crossed the CA1 cell layer. **(C)** Brain atlas section with superimposed electrode angles and positions estimated for all animals (black arrows). Arrowheads give the estimated position at which the electrodes crossed the CA1 layer. Text gives the rat number and estimated AP position of the electrode tract. Note that the brain atlas section is for an AP of -3.84mm relative to Bregma (the target AP). **(D)** Fine timescale illustration of door-pushing behaviour, showing the kernel-smoothed density estimate of number of door pushes per door side and millisecond for 100 bins per session, averaged over sessions. Profiles appear similar that on larger timescales. **(E)** Door pushes organised as in Figure 1D&E but averaged by rat instead of sessions. Statistics can be seen Table S2. Results are similar to session-averaged plots, indicating reproducibility across rats. **(F, G)** Supplement to figure in Figure 1L&M but separated by session type, averaged across sessions. Statistics can be seen in Table S2. Values are separated by distance between the current box and the goal box (distance of 1 = adjacent box). Distances of 2 are only relevant when a door is locked as both doors are optimal in open sessions and optimal distances of 3 are only available when a door was locked. No noticeable difference can be seen between the two sequence types. **(H)** Occupancy maps, i.e. average time spent in each bin, averaged across sessions for O2 and C1. Before averaging, maps were rotated so that the closed/control door was always found in the south position (in Closed-Door) or reflected around the y-axis so that all doors were locked in a clockwise direction (in One-Way). (Left) Occupancy in the Closed-Door sequence for all data (top), foraging data (middle) and goal-directed data (bottom). (Right) Same for One-Way. **(I)** (Top) Average total door push attempts per session, separated by sequence type. All pushes, whether on open or locked doors, are included. There was an effect of session but not of sequence type. With Closed-Door and One-Way combined, O2 had significantly more pushes than C2 or O3, possibly indicating decreased motivation with time. (Bottom) Same, but separated by session quarters; O3 is not shown. There was no effect of group (Closed-door vs One-Way; Table S3) so all sequence types were combined for further analysis, where a general pattern of decreasing number of door pushes with time for each session was found, except for the first session. This probably indicates a heightened exploration in the first quarter followed by decreased exploration and/or fatigue. See also Supplementary Video 1 videos of door-pushing behaviour and trajectories within the maze.

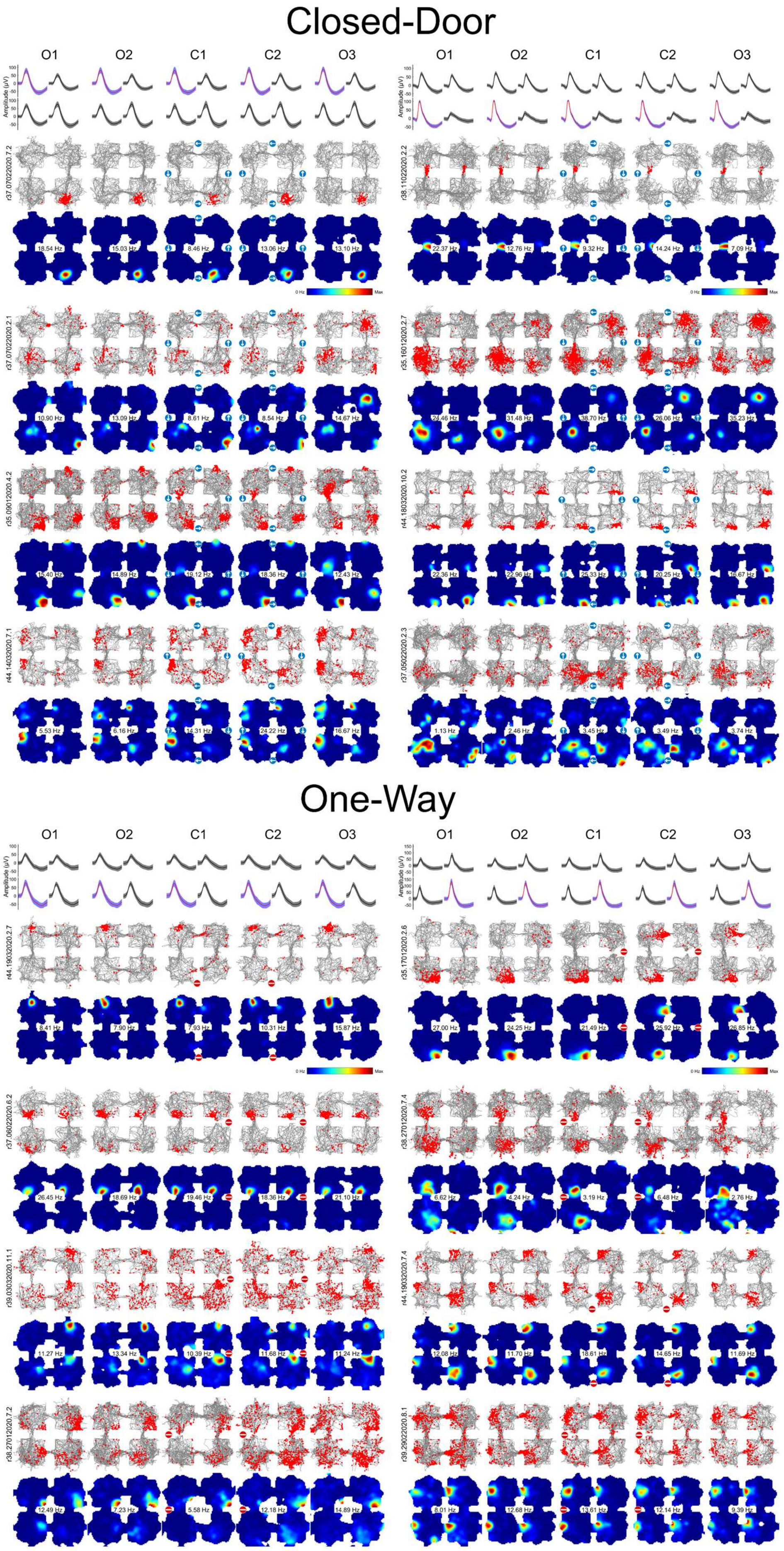

Closed-Door
O1
O2
C1
C2
O3
Amplitude (µV)
r37.07022020.7.2
18.54 Hz
15.03 Hz
8.46 Hz
13.06 Hz
13.10 Hz
r38.11022020.2.2
22.37 Hz
12.76 Hz
9.32 Hz
14.24 Hz
7.09 Hz
0 Hz
Max
r37.07022020.2.1
10.90 Hz
13.09 Hz
8.61 Hz
8.54 Hz
14.67 Hz
r35.16012020.2.7
24.46 Hz
31.48 Hz
38.70 Hz
26.06 Hz
35.23 Hz
r35.09012020.4.2
15.40 Hz
14.89 Hz
19.12 Hz
18.36 Hz
12.43 Hz
r44.18032020.10.2
22.36 Hz
22.96 Hz
25.33 Hz
20.25 Hz
16.67 Hz
r44.14032020.7.1
5.53 Hz
6.16 Hz
14.31 Hz
24.22 Hz
16.67 Hz
r37.05022020.2.3
1.13 Hz
2.46 Hz
3.45 Hz
3.49 Hz
3.74 Hz
One-Way
r44.19032020.2.7
8.41 Hz
7.90 Hz
7.93 Hz
10.31 Hz
15.87 Hz
r35.17012020.2.6
27.00 Hz
24.25 Hz
21.49 Hz
25.92 Hz
26.85 Hz
r37.06022020.6.2
26.45 Hz
18.69 Hz
19.46 Hz
18.36 Hz
21.10 Hz
r38.27012020.7.4
6.62 Hz
4.24 Hz
3.19 Hz
6.48 Hz
2.76 Hz
r39.03032020.11.1
11.27 Hz
13.34 Hz
10.39 Hz
11.68 Hz
11.24 Hz
r44.19032020.7.4
12.08 Hz
11.70 Hz
18.61 Hz
14.65 Hz
11.69 Hz
r38.27012020.7.2
12.49 Hz
7.23 Hz
5.58 Hz
12.18 Hz
14.89 Hz
r39.29022020.8.1
8.01 Hz
12.68 Hz
13.61 Hz
12.14 Hz
9.39 Hz

Figure S2: Variety of place cells responses in the Closed-Door sequence and One-Way sequence, related to Figure 2. First row: average ± standard deviation of waveform amplitudes on each tetrode channel for the first two example cells, the largest amplitude is shown in blue. Below, each row pair shows the spike plots (top: trajectory in grey, spikes in red) or speed-filtered rate maps (bottom: number indicates maximum firing rate; see *Methods - Firing rate maps and spatial information*) for a given place cell recorded in the five sessions of a sequence. Cells were selected to show a range of place field numbers (increasing from 1 at the top to 4 at the bottom) and response types across different rats. Foraging as well as goal-directed data are included. Locked doors or locked door sides are indicated by signs as in Figure 1. Cells had a variety of field numbers, repeating or not. While the spatial activity of place cells remained generally stable across consecutive sessions, place fields sometimes appeared and disappeared in a way not specifically linked to the changes in connectivity. See also Supplementary Video 2 for spike plots and rate maps of all used place cells (foraging + goal-directed).

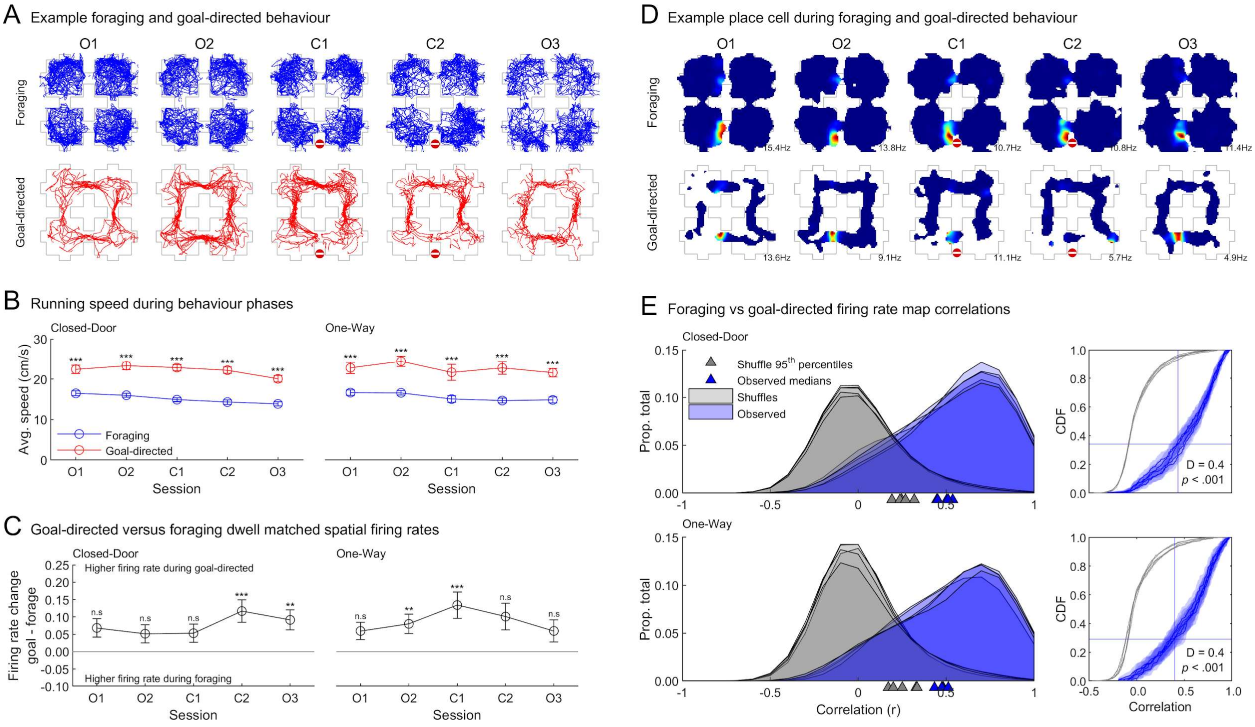

Figure S3: No spatial remapping between foraging and goal-directed epochs, related to Figure 2. See Table S4 for all statistical results. Task remapping has been shown under some conditions, usually if tasks are performed in separate blocks [S1-3], but not when tasks are intermingled within the same experimental session [S4-5]. We compared foraging to goal-directed activity for each session type, see *Methods - Behaviour discrimination*. **(A)** Example of trajectory discrimination between foraging (Top) and goal-directed (Bottom) for one Closed-Door sequence. Note that foraging trajectories are comparable between sessions while goal-directed ones are strongly affected by the door closure; see also Figure S1H for dwell maps averaged over all sessions. **(B)** Average running speed over sessions for the different task phases. Speed during goal-directed movement was always higher. Speed was slower in later sessions, probably indicative of decreasing motivation. **(C)** Comparison of average firing rate between task epochs, computed over common spatial bins as the difference of goal-directed and foraging average firing rates, averaged over place cells. Positive numbers indicate increased firing rate during goal-directed epochs, significantly so for a subset of the sessions (two-sided signed rank tests with Holm-Bonferroni correction). **(D)** Example rate maps of a cell recorded in the same session as A, divided between foraging (Top) and goal-directed (Bottom). Note that the spatial activity of this cell remains stable between the two types of behaviours. Colour scale is the same as previous rate maps, number indicates peak firing rate. See *Methods - Foraging vs goal-directed remapping.* **(E)** (Left) Distributions of the correlations between goal-directed and foraging rate maps for all sessions (O1, O2, C1, C2, O3) in blue and shuffled data in grey. In all sessions, the median of the observed correlations exceeded the 95th percentile of a corresponding chance distribution, demonstrating that the data from both task phases were more similar than chance, indicating no global remapping between the two task phases. (Right) Cumulative distribution function of each group. The 95th percentile of the shuffle distribution (O2) is indicated by a vertical blue line and the intersection of this with the O2 data distribution is indicated by a horizontal line, showing that more than 60% of the cells are more stable than chance; thus, only a minority of cells changed their firing location between foraging and goal-directed phases. (<37% in Closed-Door, <30% in One-Way). Text gives the result of a two-sample Kolmogorov-Smirnov test comparing the two distributions in session O2. There was no effect of connectivity on this (no significant difference between data distributions, all p>.05, Kolmogorov-Smirnov tests, Holm-Bonferroni corrected).

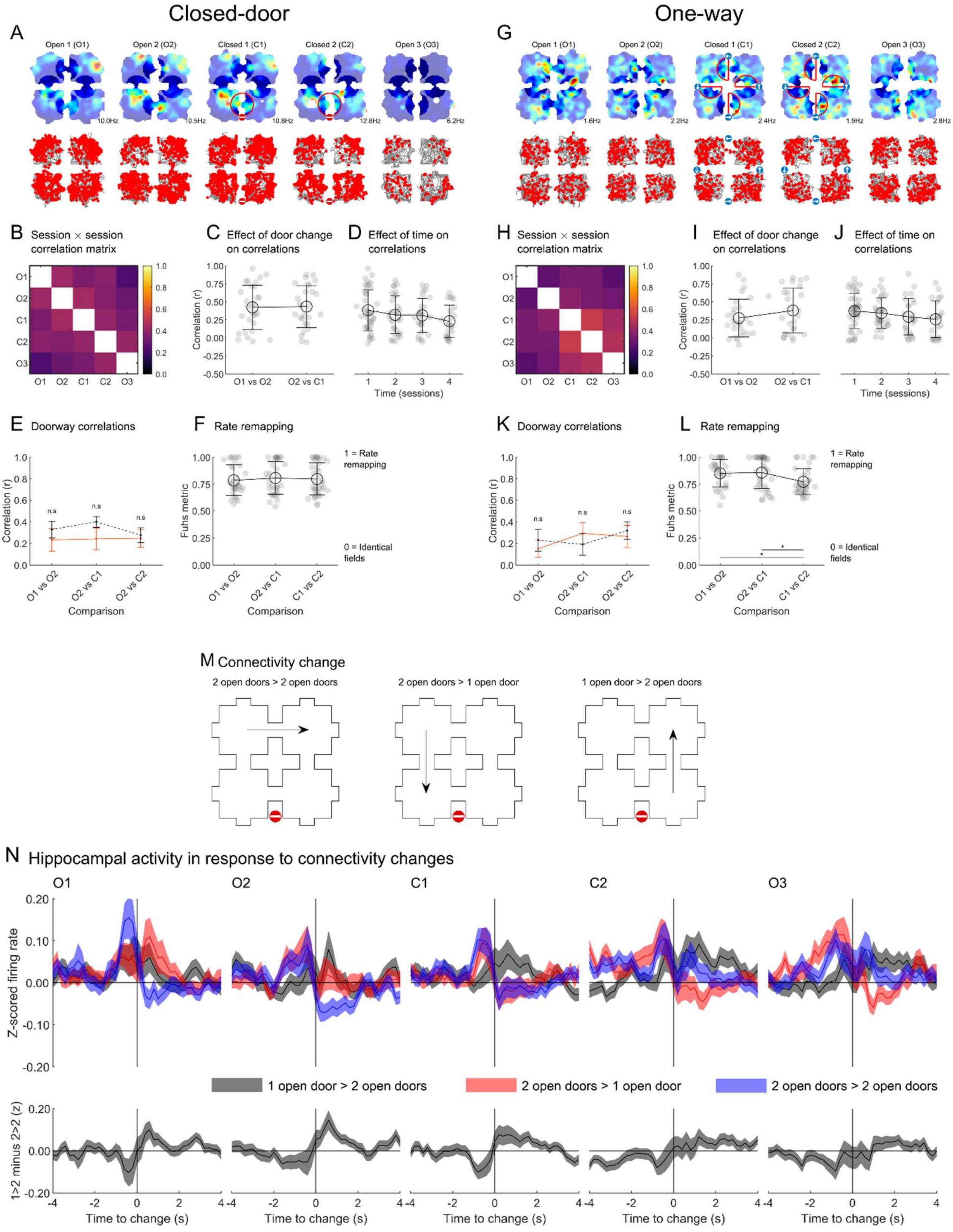

Figure S4: No effect of connectivity changes on pyramidal non-place cells or on average place cell firing rate when crossing doors, related to Figure 2. (**A-L**) Pyramidal non-place cells were analysed in the same was as place cells in Figure 2, with A-F related to the Closed-Door sequence and G-L related to One-Way, legend is the same as Figure 2. (**C**) No effect of session was found, similarly to place cells: $F(1,54) = 0.0$, $p = .89$. (**D**) No effect of time was found, unlike for place cells $F(3,115) = 1.7$, $p = .16$. (**F**) There was no effect of connectivity on rate remapping: $F(2,98) = 0.2$,

p = .85. (**I**) There was no effect of session on cross-session correlations: F(1,51) = 1.8, p = .19. (**J**) Again, unlike for place cells, no effect of time on correlations was found: F(3,118) = 1.4, *p* = .26; (**L**) An effect of session was found for rate remapping: F(2,102) = 4.5*, p* = .014; post-hoc tests are as follows: O1O2 vs O2C1: *p* = .97; O1O2 vs C1C2: *p* = .047; O2C1 vs C1C2: *p* = .019; however, these effects are not specific to the connectivity change. In summary, even in this population of non-place cells, no effect specific to changing the connectivity (i.e. when comparing O2 to C1) was found. Interestingly, the effect of time observed in place cells was not significant in this population of non-place cells. In addition, average cross-session correlation values were generally lower than for place cells (Figure 2), indicating less spatially-stable activity in this population. (**M, N**) Human participants entering a street that had greater connectivity than the preceding street had increased activity in their posterior right hippocampus, which decreased if the street had less connectivity [S6]. We tested this here by comparing activity when entering a box depending on the number of transitions available from that box. Z-scores of firing rates were computed for each place cell, binned in 0.2s bins in a time window ranging from -4s to +4s around entry into a box, and averaged over all place cells. No effect was found. (**M**) Schematic showing the possible connectivity transitions available to the rats when one door is closed. Rats can move to a box with equivalent connectedness (Left), diminished connectedness (Middle) or increased connectedness (Right). (**N**) (Top) Mean and SEM firing rates for all place cells around these connectivity transitions, using all data (foraging + goal-directed). (Bottom) Difference between the firing rate profile for increased connectedness and equivalent connectedness. Values higher than 1.96 or lower than -1.96 would represent a significant change in activity. Note that in the open sessions (O1, O2 & O3) all compartments are equally connected so the groups are arbitrary.

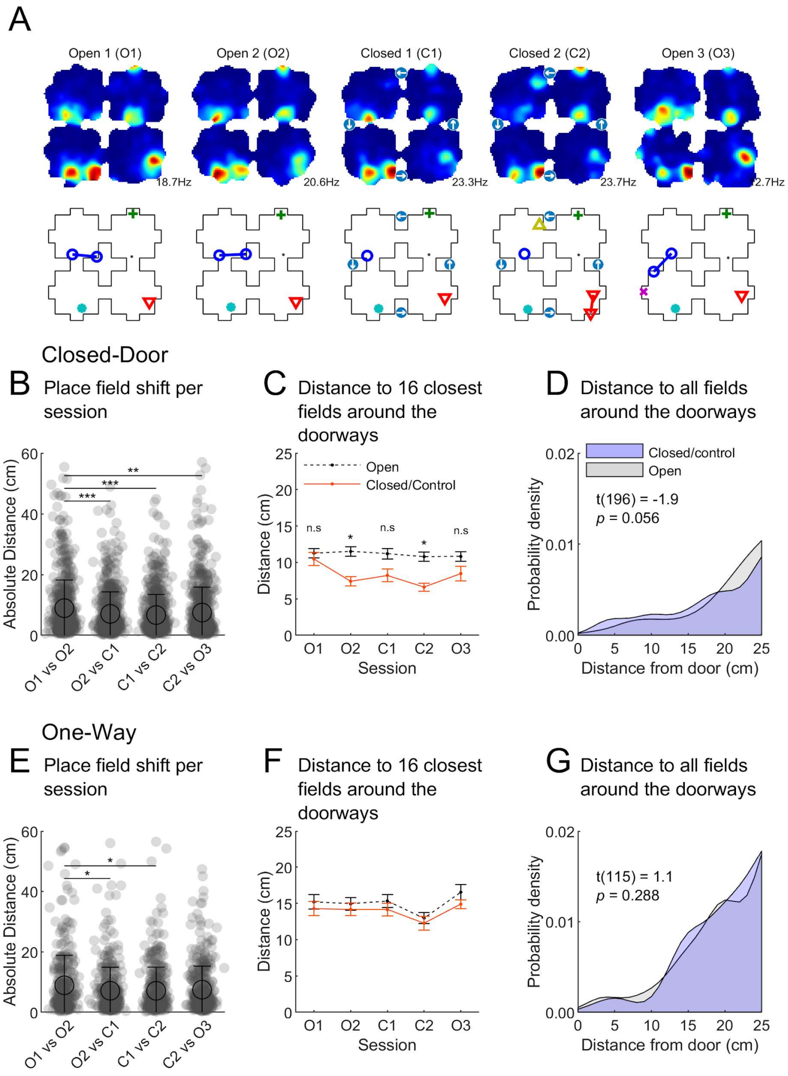

Figure S5: No change in distance of fields to doors with connectivity, related to Figure 3. See Table S4 for statistics and *Methods – Fields distance to doorways*. (**A**) An example place cell recorded across sessions. (Top) Firing rate maps, blue denotes low or no firing, red denotes high firing. Maximum firing rates are given as text. (Bottom) Centroids of place fields detected in each map. Each field is tracked across sessions and represented by the same coloured/shaped marker in each. Where a field was detected to split in one or more sessions, the same marker is joined by a line of the same colour. (**B**) Absolute distance that place field centroids shifted of between consecutive sessions. Place fields moved the most between sessions O1 and O2 indicating no specific field movement due to connectivity change. (**C**) Distance to the closest 16 fields from the doors in each closed-door session. In O2 and C2, fields are significantly closer to the locked doors, although this is a general trend present in almost all sessions, including O2 and O3 where there was no connectivity change. Overall, although there were some differences between the control/locked door and the open doors, these effects were not specific to the closed-door sessions. (**D**) Distance to all fields from the doors in closed-door session C1 only. Text gives the result of a two-sample t-test comparing the distributions. These were not significantly different. (**E-G**) Same as B-D but for the One-Way condition with similar results.

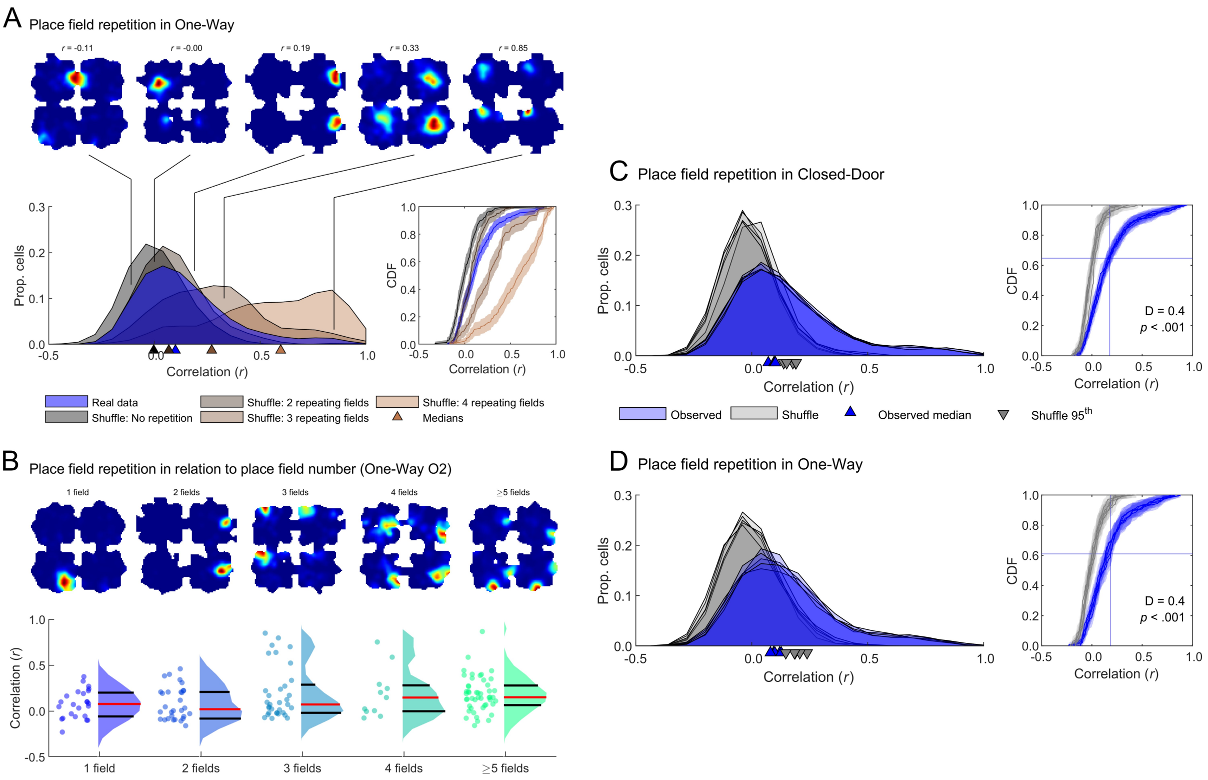

Figure S6: Most place cells encode space globally in One-Way too, independently of connectivity. Related to Figure 4. (**A&B**) Supplement for Figure 4D, E showing data for the One-Way condition; see *Methods - Place field repetition*. (**A**) (Top) Example rate maps (all data, speed-filtered, red indicates high firing rate, dark blue indicates low firing rate) with indicated correlation values on the O2 data distribution (blue). (Left) Blue distribution represents real data cross-box correlation values for session O2. All other distributions represent different shuffles designed to simulate the correlations expected if cells did not have any repeating fields or repeated fields in 2, 3 or 4 compartments. Medians for all distributions are indicated by corresponding markers along the x-axis. (Right) Same data as left but showing the empirical cumulative distribution functions, shaded areas represent lower and upper confidence intervals. Results are similar to Closed-Door with average cross-box correlation values most similar to a situation with 2 repeating fields. (**B**) Same as Figure 4E for One-Way (O2 only). (Top) Example rate maps sorted by the number of detected place fields. Bottom: box correlations scores also separated by number of detected fields; dots show individual place cell data, distribution is shown as violin plot, with median in red and quartiles in black. The same bimodality as for Closed-Door emerges for 3 or more fields, showing a small subset of cells with fully-repeating place fields (correlations>0.5). (**C**) (Left) In blue, distributions of cross-box correlation values for all sessions (O1, O2, C1, C2, O3); in grey, distributions of shuffled correlation values for each session. Medians for all distributions are indicated by corresponding markers along the x-axis. Data medians were always below the 95th percentile of the corresponding shuffle, indicating similarity to the shuffle, i.e. low field repetition overall. (Right) Same data but showing the empirical cumulative distribution functions, shaded areas represent lower and upper confidence intervals. Vertical blue line indicates the 95th percentile of the shuffle distribution (for O2) and its intersection with the data distribution is indicated by a horizontal line, showing that the majority of cells do not repeat more than chance. Text gives the result of a two-sample Kolmogorov-Smirnov test comparing the two distributions for O2. Data distributions from different session types did not differ from each other (p>.05 in all cases, two-sample Kolmogorov-Smirnov tests), in line with our other results finding no effect of connectivity changes. (**D**) Same as C but for One-Way.

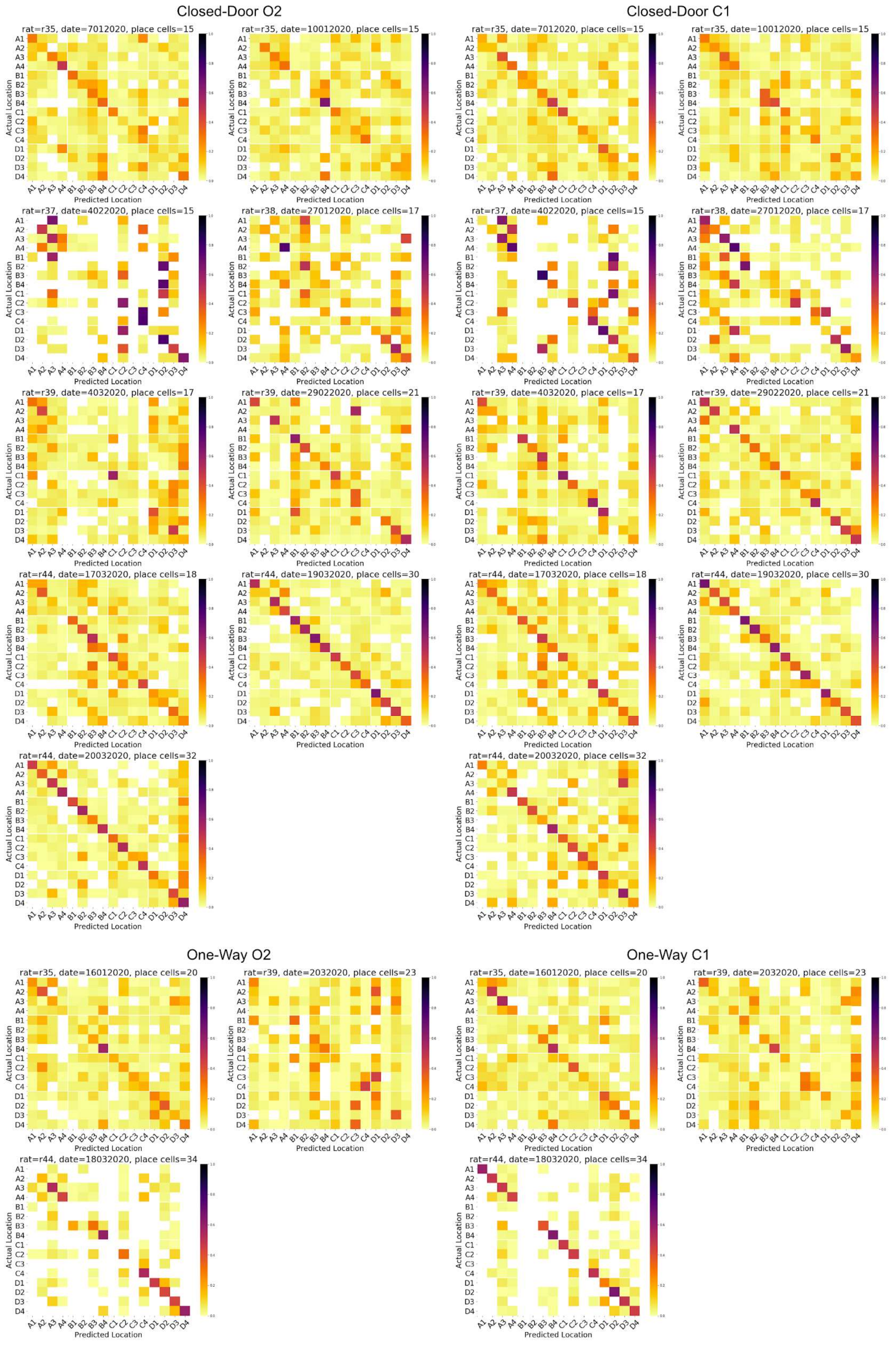

Figure S7: Individual confusion matrices for all sessions used in the quadrant Bayesian decoding analysis, related to Figure 4F-H. See *Methods - Bayesian decoding of box quadrants.* Only sessions with at least 15 simultaneously recorded place cells were used. The diagonal pattern can be observed for most sessions, with some exceptions for those with lower numbers of cells (indicated in the title of each plot).

	Number of full sequences		Place cells (% of all neurons)		Place fields	
Rat	Closed-Door	One-Way	Closed-Door	One-Way	Closed-Door	One-Way
r35	6	3	73 (74.5)	43 (75.4)	525	267
r37	3	2	29 (80.6)	26 (83.9)	91	95
r38	4	2	32 (94.1)	12 (70.6)	93	53
r39	3	2	47 (79.7)	35 (58.3)	254	358
r44	3	2	80 (81.6)	45 (67.2)	412	236
Total	19	11	261 (80.3)	161 (69.4)	1375	1009

Table S1: Numbers of sequences, place cell and place field numbers per rat and condition, related to Figure 1 and 2. Note that place field numbers used the session with the highest mean firing rate (used for place cell categorisation), thus, they represent an upper bound and are not representative of average sessions.

Condition	Isolation distance	L ratio	Waveform height (µV)	Waveform width (µs)	Mean firing rate (Hz)
Closed-Door	23.6 ± 24.2	0.03 ± 0.06	48.4 ± 39.5	485.1 ± 65	0.73 ± 0.76
One-way	20.2 ± 13.4	0.04 ± 0.1	45.5 ± 32.4	481.9 ± 73	0.65 ± 0.63

Table S2: Cluster quality measures for putative place cells, related to Figure 2. See *Methods - Cluster quality measures* for detailed methods regarding these metrics. Values are mean ± standard deviation

Panel and dependent variable	Omnibus test	Effect of group (E-G: closed vs open; I: Closed-Door or One-Way)	Effect of session (O1, O2, C1 …) or quarters (I with sequences combined)	Interaction groupXsession or group X quarters (I bottom)	Post-hoc tests across groups (corrected)
E<br>Closed-Door, pushes/door/min	Two-way repeated measures ANOVA	$F(1,16) = 13.5$, $p = .021$	$F(4,16) = 5.3$, $p = .006$	$F(4,16) = 27.1$, $p < .001$	C1: $p < .001$<br>C2: $p < .001$<br>All other $p > .90$
E<br>One-Way, pushes/door/min		$F(1,16) = 20.2$, $p = .011$	$F(4,16) = 3.2$, $p = .041$	$F(4,16) = 26.4$, $p < .001$	C1: $p < .001$<br>C2: $p < .001$<br>O3: $p = .006$<br>All other $p > .90$
F<br>Correct first door choices post-bell, Closed-Door	Independent one-sample t-tests with correction	Compared to chance (1/2):<br>From left to right: $p > .99$, $p = .003$, $p < .001$, $p < .001$, $p > .99$, $p > .99$, $p > .99$, $p > .99$, $p > .99$			
F<br>Correct first door choices post-bell, One-Way		Compared to chance (1/2):<br>From left to right: $p > .99$, $p > .99$, $p > .99$, $p = .004$, $p > .99$, $p < .001$, $p = .001$, $p > .99$, $p > .99$			
G<br>Correct first foraging post-bell, Closed-door		Compared to chance (1/3):<br>From left to right: $p < .001$, $p < .001$, $p < .001$, $p > .99$, $p < .001$, $p > .99$, $p > .99$, $p < .001$, $p > .99$, $p > .99$, $p < .001$, $p > .99$			
G<br>Correct first foraging post-bell, One-Way		Compared to chance (1/3):<br>From left to right: $p = .003$, $p > .99$, $p < .001$, $p > .99$, $p < .001$, $p > .99$, $p > .99$, $p < .001$, $p > .99$, $p > .99$, $p < .001$, $p > .99$			
I (Top)<br>Total number of pushes	Two-way repeated measures ANOVA	$F(1,140) = 0.7$, $p=.398$	$F(4,140) = 2.5$, $p=.043$	$F(4,140) = 0.1$, $p=.98$	All $p > .90$
I (Top)<br>Total number of pushes, Closed-Door and One-Way combined	One-way repeated measures ANOVA	N.A.	$F(4,145) = 2.8$, $p=.026$	N.A.	O1 vs O3: $p=.048$<br>C1 vs O3: $p=.041$<br>All other $p>.05$
I (Bottom)<br>Total number of pushes, O1	Two-way repeated measures ANOVA	$F(1,112) = 0.9$, $p = .34$	$F(3,112) = 1.4$, $p = 0.25$	$F(3,112) = 0.3$, $p = .84$	Effect of group in each quarter:<br>All $p > .90$
I (Bottom)<br>Total number of pushes, O2		$F(1,112) = 0.1$, $p = .71$	$F(3,112) = 4.9$, $p = .0031$	$F(3,112) = 0.4$, $p = .73$	Effect of group in each quarter:<br>All $p > .90$
I (Bottom)<br>Total number of pushes, C1		$F(1,112) = 0.0$, $p = .91$	$F(3,112) = 20.4$, $p < .001$	$F(3,112) = 0.4$, $p = .72$	Effect of group in each quarter:<br>All $p > .90$
I (Bottom)<br>Total number of pushes, C2		$F(1,112) = 0.0$, $p = .90$	$F(3,112) = 10.8$, $p < .001$	$F(3,112) = 0.4$, $p = .74$	Effect of group in each quarter:<br>All $p > .90$
I (Bottom)<br>Total number of pushes, Closed-Door and One-Way combined, O1	One-way repeated measures ANOVA	N.A.	$F(3,116) = 1.2$, $p = 0.31$	N.A.	N.A.
I (Bottom)<br>Total number of pushes, Closed-Door and One-Way combined, O2		N.A.	$F(3,116) = 5.5$, $p < .001$	N.A.	Q1 vs Q4: $p<.001$<br>all other $p > .05$
I (Bottom)<br>Total number of pushes, Closed-Door and One-Way combined, C1		N.A.	$F(3,116) = 3.5$, $p < .001$	N.A.	Q1 vs Q2,<br>Q1 vs Q3,<br>Q1 vs Q4:<br>$p < .001$<br>all other $p > .50$
I (Bottom)<br>Total number of pushes, sequences combined, C2		N.A.	$F(3,116) = 12.1$, $p < .001$	N.A.	Q1 vs Q2: $p=.0033$<br>Q1 vs Q3: $p<.001$<br>Q1 vs Q4: $p<.001$<br>all other $p>.10$

Table S3: Statistics corresponding to Figure S1. All post-hoc tests were based on estimated marginal means and corrected using the Tukey-Kramer method.

Panel and dependent variable	Omnibus test	Effect of group (closed/control or open)	Effect of session	Interaction group X session	Post-hoc tests across groups (corrected)
B left<br>Foraging vs goal-directed running speed Closed-Door	two-way repeated measures ANOVA	$F(1,72) = 156.2$, $p < .001$	$F(4,72) = 6.8$, $p < .001$	$F(4,72) = 4.2$, $p = 0.042$	All $p < .001$
B right<br>Foraging vs goal-directed running speed One-Way	two-way repeated measures ANOVA	$F(1,40) = 47.5$, $p < .001$	$F(4,40) = 3.9$, $p = 0.0089$	$F(4,40) = 1.2$, $p = 0.32$	All $p < .001$
C left<br>Foraging vs goal-directed firing rates One-Way	Independent one-sample t-tests with Holm-Bonferroni correction	Compared to chance (0):<br>From left to right: $p > .99$, $p > .99$, $p > .99$, $p < .001$, $p = .002$			
C right<br>Foraging vs goal-directed firing rates One-Way		Compared to chance (0):<br>From left to right: $p > .99$, $p = .004$, $p < .001$, $p > .99$, $p > .99$			

Table S4: Statistical test results corresponding to Figure S3. Note: all post-hoc tests were based on estimated marginal means and corrected using the Tukey-Kramer method.

Supplemental References

S1. Markus, E.J., Qin, Y.L., Leonard, B., Skaggs, W.E., McNaughton, B.L., and Barnes, C.A. (1995). Interactions between location and task affect the spatial and directional firing of hippocampal neurons. J. Neurosci. *15*, 7079–7094.

S2. Wiener, S.I., Paul, C.A., and Eichenbaum, H. (1989). Spatial and behavioral correlates of hippocampal neuronal activity. J. Neurosci. *9*, 2737–2763.

S3. Spiers, H.J., Olafsdottir, H.F., and Lever, C. (2018). Hippocampal CA1 activity correlated with the distance to the goal and navigation performance. Hippocampus *28*, 644–658.

S4. Trullier, O., Shibata, R., Mulder, A.B., and Wiener, S.I. (1999). Hippocampal neuronal position selectivity remains fixed to room cues only in rats alternating between place navigation and beacon approach tasks. Eur. J. Neurosci. *11*, 4381–4388.

S5. Duvelle, É., Grieves, R.M., Hok, V., Poucet, B., Arleo, A., Jeffery, K.J., and Save, E. (2019). Insensitivity of Place Cells to the Value of Spatial Goals in a Two-Choice Flexible Navigation Task. J. Neurosci. *39*, 2522–2541.

S6. Javadi, A.-H., Emo, B., Howard, L.R., Zisch, F.E., Yu, Y., Knight, R., Pinelo Silva, J., and Spiers, H.J. (2017). Hippocampal and prefrontal processing of network topology to simulate the future. Nat. Commun. *8*, 14652.
